# The role of chloroplast SRP54 domains and its C-terminal tail region in post- and co-translational protein transport *in vivo*

**DOI:** 10.1093/jxb/erae293

**Published:** 2024-07-11

**Authors:** Annika Bischoff, Jennifer Ortelt, Beatrix Dünschede, Victor Zegarra, Patricia Bedrunka, Gert Bange, Danja Schünemann

**Affiliations:** Molecular Biology of Plant Organelles, Ruhr University Bochum, Bochum, Germany; Molecular Biology of Plant Organelles, Ruhr University Bochum, Bochum, Germany; Molecular Biology of Plant Organelles, Ruhr University Bochum, Bochum, Germany; Center for Synthetic Microbiology (SYNMIKRO) and Department of Chemistry, University of Marburg, Marburg, Germany; Center for Synthetic Microbiology (SYNMIKRO) and Department of Chemistry, University of Marburg, Marburg, Germany; Center for Synthetic Microbiology (SYNMIKRO) and Department of Chemistry, University of Marburg, Marburg, Germany; Max-Planck-Institute for terrestrial Microbiology, Marburg, Germany; Molecular Biology of Plant Organelles, Ruhr University Bochum, Bochum, Germany; Bielefeld University, Germany

**Keywords:** Arabidopsis, BN-PAGE, chloroplast, cpSRP54, photosystem I assembly, protein transport, signal recognition particle, thylakoid membrane

## Abstract

In the chloroplast, the 54 kDa subunit of the signal recognition particle (cpSRP54) is involved in the post-translational transport of the light-harvesting chlorophyll *a*/*b*-binding proteins (LHCPs) and the co-translational transport of plastid-encoded subunits of the photosynthetic complexes to the thylakoid membrane. It forms a high-affinity complex with plastid-specific cpSRP43 for post-translational transport, while a ribosome-associated pool coordinates its co-translational function. CpSRP54 constitutes a conserved multidomain protein, comprising a GTPase (NG) and a methionine-rich (M) domain linked by a flexible region. It is further characterized by a plastid-specific C-terminal tail region containing the cpSRP43-binding motif. To characterize the physiological role of the various regions of cpSRP54 in thylakoid membrane protein transport, we generated Arabidopsis cpSRP54 knockout (*ffc1-2*) lines producing truncated cpSRP54 variants or a GTPase point mutation variant. Phenotypic characterization of the complementation lines demonstrated that the C-terminal tail region of cpSRP54 plays an important role exclusively in post-translational LHCP transport. Furthermore, we show that the GTPase activity of cpSRP54 plays an essential role in the transport pathways for both nuclear as well as plastid-encoded proteins. In addition, our data revealed that plants expressing cpSRP54 without the C-terminal region exhibit a strongly increased accumulation of a photosystem I assembly intermediate.

## Teaser

The chloroplast signal recognition particle subunit cpSRP54 has an important role in the biogenesis of both the light harvesting antenna complex and the cores of photosystem I and II.

## Introduction

The light-harvesting chlorophyll *a*/*b*-binding proteins (LHCPs) are the most abundant integral proteins of the thylakoid membrane and play a pivotal role in capturing and channeling solar energy to the photosystem (PS) I and II reaction centers. In Arabidopsis, the PSII antenna (light-harvesting antenna complex II, LHCII) is mainly formed by the LHCP family members Lhcb1–6, while the proteins Lhca1–4 are the major constituents of the PSI antenna (light-harvesting antenna complex I, LHCI) (Ben-Shem *et al.*, 2003; Nelson and Ben-Shem, 2004; Mazor *et al.*, 2017; van Bezouwen *et al.*, 2017). The biogenesis of the nuclear-encoded LHCPs requires a series of orchestrated steps to ensure proper targeting and integration into the thylakoid membrane.

After import of the LHCP through the TOC/TIC translocation machinery of the chloroplast envelope, it is bound by the post-translationally acting stromal chloroplast signal recognition particle (cpSRP) (Akopian *et al.*, 2013; Ziehe *et al.*, 2018). CpSRP is a high affinity heterodimer formed by the 43 kDa and 54 kDa subunits cpSRP43 and cpSRP54, respectively (Franklin and Hoffman, 1993; Schünemann *et al.*, 1998; Klimyuk *et al.*, 1999; Gao *et al.*, 2015; Ziehe *et al.*, 2018). The LHCP–cpSRP complex (transit complex) docks to the thylakoid membrane through an interaction with the cpSRP54 receptor, cpFtsY, and the Alb3 insertase (Kogata *et al.*, 1999; Tu *et al.*, 1999; Moore *et al.*, 2000; Asakura *et al.*, 2008). *In vitro* experiments demonstrated that the insertion of LHCP into the membrane requires GTP (Hoffman and Franklin, 1994; Yuan *et al.*, 2002). GTP triggers the GTPase cycle of the cpSRP54–cpFtsY complex as both proteins interact directly by their GTPase-coding NG-domains, which stimulates each other’s hydrolysis activity and dissociation of the complex (Jaru-Ampornpan *et al.*, 2007, 2009). A central step in LHCP transport is the accommodation of its hydrophobic domains by cpSRP to maintain its solubility and insertion competence. Here, cpSRP43 plays a key role, as it interacts directly with a conserved DPLG motif between the second and third transmembrane helix of LHCP and is sufficient to prevent LHCP from aggregation (DeLille *et al.*, 2000; Tu *et al.*, 2000; Falk and Sinning, 2010; Jaru-Ampornpan *et al.*, 2010). Furthermore, it has been suggested that LHCPs can be sorted to the thylakoid membrane by cpSRP43 alone at least in an Arabidopsis double mutant lacking cpSRP54 and cpFtsY (Tzvetkova-Chevolleau *et al.*, 2007). The role of cpSRP54 in transit complex formation is less defined. While some data indicate that cpSRP54 plays an indirect role in transit complex formation by driving cpSRP43 into an active state that allows tight binding to LHCPs, other data point to a direct binding of cpSRP54 to the third transmembrane helix of LHCP (High *et al.*, 1997; Groves *et al.*, 2001; Henderson *et al.*, 2016; Liang *et al.*, 2016). The interaction between cpSRP54 and cpSRP43 has been extensively investigated, highlighting its dependence on the positively charged ‘ARRKR’ motif situated in the C-terminal tail region of cpSRP54. This motif facilitates binding to the second chromodomain of cpSRP43 (Funke *et al.*, 2005; Holdermann *et al.*, 2012; Dünschede *et al.*, 2015).

**Fig. 1.**
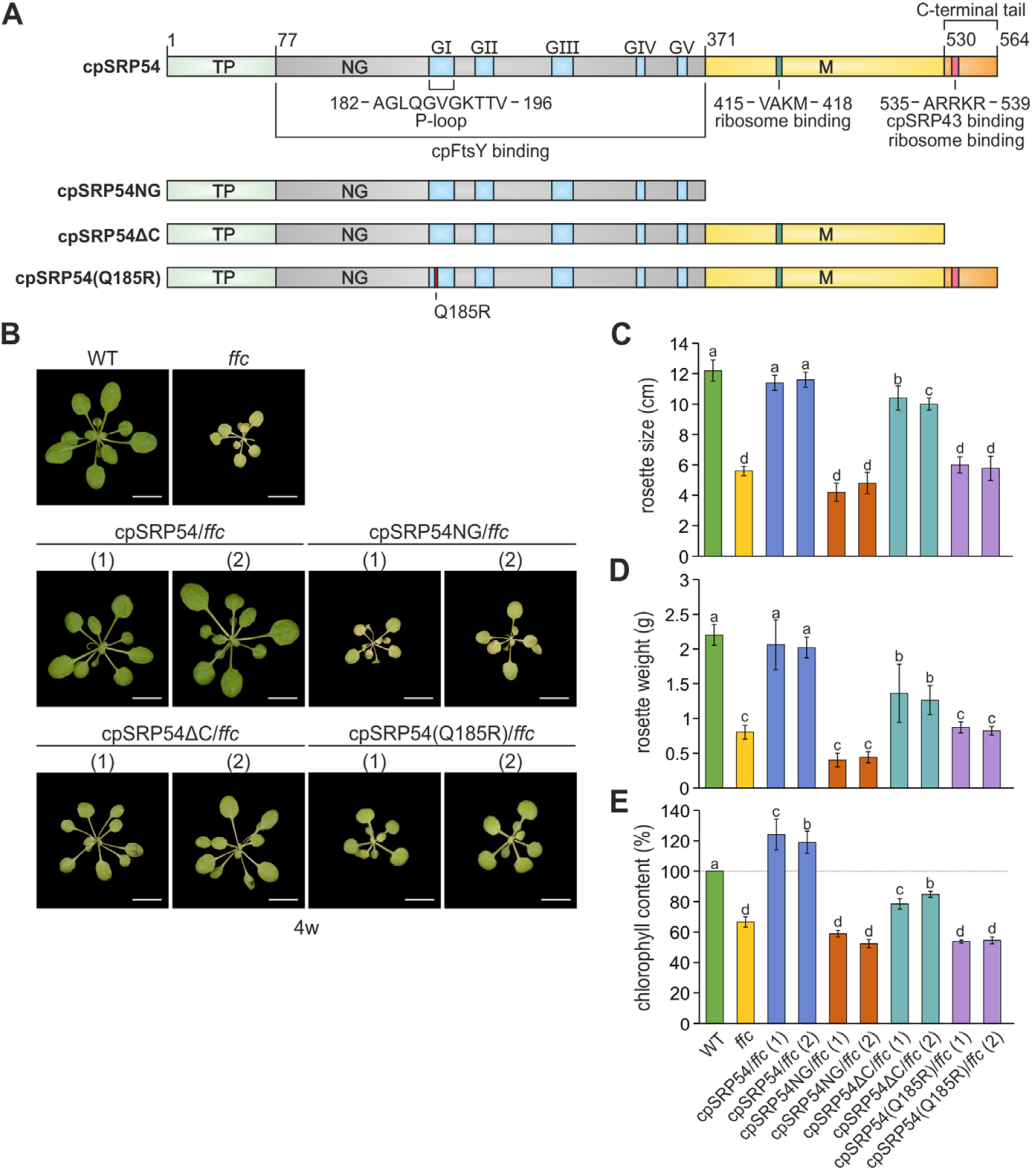
Arabidopsis cpSRP54 variants and complementation lines of *ffc*. (A) Scheme for the Arabidopsis cpSRP54 protein and variants used to generate *ffc*-complementation lines. The multidomain protein cpSRP54 is composed of an amino-terminal N-domain in close contact with a G-domain (NG), a methionine-rich M-domain, and a C-terminal tail region. The G-domain provides the interaction region of cpSRP54 and its receptor cpFtsY including the conserved GTPase motifs GI (phosphate (P)-binding loop)–GV. The cpSRP43 binding motif, which serves also as one of the two ribosomal binding sites is located within the C-terminal tail region. The second ribosomal binding motif is located within the M-domain. Schematic representation according to Ziehe *et al.* (2018). (B) Phenotypes of 4-week-old Arabidopsis *ffc*-complementation lines compared with wild type (WT) and the cpSRP54-lacking mutant *ffc*. The scale bar (white line) corresponds to 1 cm. The Arabidopsis *ffc*-complementation lines display altered phenotypes. (C, D) The rosette size (C) and rosette weight (D) was measured from 10-week-old plants. (E) The chlorophyll content was determined from 4-week-old Arabidopsis plants in relation to wild type (WT, dotted line, 100%). In each analysis the average values and corresponding standard deviations were calculated from three to eight independent experiments with independently grown plants. Different letters indicate statistically significant differences (*P*<0.05) between means determined by one-way ANOVA followed by Dunnett’s multiple comparisons test.

Remarkably, a secondary pool of stromal cpSRP54 is linked with chloroplast ribosomes, playing a crucial role in facilitating the efficient co-translational sorting of plastid-encoded multi-span thylakoid membrane proteins. These include components like the reaction center subunits of photosystem I (PsaA, PsaB) and photosystem II (PsbA, PsbD) (Franklin and Hoffman, 1993; Schünemann *et al.*, 1998; Hristou *et al.*, 2019). The ribosomal binding interface on cpSRP54 has been assigned to two binding motifs, one corresponding to the ARRKR motif in the C-terminal tail and the second formed by a short motif within the M-domain of cpSRP54 (Hristou *et al.*, 2019) (Fig. 1A). Furthermore, a direct contact of cpSRP54 with a hydrophobic region of the nascent PsbA chain has been demonstrated (Nilsson *et al.*, 1999; Nilsson and van Wijk, 2002). While the precise molecular mechanism governing the targeting and insertion of nascent chains into the thylakoid membrane remains largely unresolved, current data indicate that a contact between cpSRP54 and cpFtsY facilitates the docking of the translating ribosome to the thylakoid membrane. Subsequently, membrane insertion is thought to be mediated by the cpSec1/Alb3 insertion machinery (Zhang *et al.*, 2001; Göhre *et al.*, 2006; Tzvetkova-Chevolleau *et al.*, 2007; Walter *et al.*, 2015a, b).

This study was performed to analyse the physiological role of the individual domains of cpSRP54 in post- and co-translational protein sorting *in vivo*. We transformed *ffc1-2* Arabidopsis plants (lacking cpSRP54) with cpSRP54 constructs encoding C-terminal truncations or the point mutation variant cpSRP54(Q185R), which is impaired in GTP hydrolysis activity. Our data show that the plant-specific C-terminal tail region of cpSRP54 is required for post-translational transport of the LHCPs *in vivo*. In addition, we provide *in vivo* evidence that the GTPase activity of cpSRP54 is essential for post-translational LHCP sorting as well as for co-translational insertion. Our study also shows the accumulation of a PSI assembly intermediate in the *ffc1-2* line expressing a cpSRP54 variant, which lacks its C-terminal extension. Furthermore, our data indicate that the assembly of LHCI to PSI is required for the stable incorporation of PsaK into PSI.

## Materials and methods

### Generation of Arabidopsis complementation lines

To generate complementation lines, Arabidopsis *ffc* mutant plants [*ffc1-2*, previously described in Amin *et al.* (1999)] were transformed with the plant transformation vector pCBi18 (Biesgen and Weiler, 1999; Lehmann *et al.*, 2017) containing cDNA fragments of Arabidopsis cpSRP54 (Accession number: At5g03940) variants. The full-length coding sequence of Arabidopsis cpSRP54 was obtained from the Arabidopsis biological resource center (ABRC Stock Number: U10271). The residues 1–564 (At-cpSRP54), 1–370 (At-cpSRP54NG), and 1–529 (At-cpSRP54∆C) were introduced downstream the CaMV 35S promoter into pCBi18, replacing the originally included *uidA* gene via *Xba*I/*Sac*I restriction sites. For plasmid construction, gene-specific primers adding a His-tag sequence were used with the In-Fusion HD EcoDry kit (Takara). The complementation construct containing Arabidopsis cpSRP54 residues 1–564 including a point mutation at position 185 (At-cpSRP54(Q185R)) was generated using the QuikChange Lightning site-directed mutagenesis kit (Agilent Technologies) using pCBi18-At-cpSRP54 as template. The primers used are listed in Supplementary Table S1. All plasmids were checked by Sanger sequencing and used for *Agrobacterium tumefaciens*-mediated plant transformation (De Block, 1993; Chateau *et al.*, 2000). The plasmids were introduced into *Agrobacterium tumefaciens* GV3101 by electroporation and subsequently transformed into Arabidopsis *ffc* background by floral dip (Clough and Bent, 1998). The homozygous plant lines were selected as described in Lehmann *et al.* (2017) and identified via genotyping PCR (Supplementary Fig. S1A). All PCR products were checked by Sanger sequencing. The primers used are listed in Supplementary Table S1. The transgene expression was additionally verified by western blot (Supplementary Fig. S1B).

### Plant material, growth conditions and phenotypic analyses

Arabidopsis wild type (Columbia-0, Col-0), *ffc* (*ffc1-2*, previously described in Amin *et al.* (1999), and generated complementation lines (cpSRP54/*ffc*, cpSRP54NG/*ffc*, cpSRP54∆C/*ffc*, cpSRP54(Q185R)/*ffc*) were grown on soil under artificial light (Philips Master TL-D 58W/840 Reflex Eco: 8 h light, 120 µmol m^−2^ s^−1^, 22 °C, 65% humidity; 16 h dark, 19.5 °C, 65% humidity). Total chlorophyll content of 100 mg 4-week-old Arabidopsis leaves was determined using 80% (v/v) acetone according to Porra *et al.* (1989). The rosette size (diameter) and weight were measured from 10-week-old plants.

### Genomic DNA extraction and genotyping PCR

Genomic DNA was extracted from two to three small Arabidopsis leaves of Col-0 (WT), *ffc*, cpSRP54/*ffc*, cpSRP54NG/*ffc*, cpSRP54∆C/*ffc*, and cpSRP54(Q185R)/*ffc*. The resulting gDNA pellet was air dried and dissolved in 40 µl TE buffer (10 mM Tris pH 8.0, 5 mM EDTA pH 8.0) followed by incubation for 15 min at 55 °C with rotation (350 rpm). Unresolved impurities were sedimented [18 500 *g*, room temperature (RT), 5 min], and the gDNA-containing supernatant was used for genotyping PCR by using the GoTaq Polymerase (Promega). The primers used are listed in Supplementary Table S1.

### Measurement of the maximum quantum yield of photosystem II

The maximum quantum yield of PSII (*F*_v_/*F*_m_) was analysed using the Dual-PAM-100 measuring system (Walz GmbH, Germany). The 8-week-old Arabidopsis plants were acclimated in the dark for 30 min prior to measurement. *F*_v_/*F*_m_ was determined after either normal light (120 µmol m^−2^ s^−1^) or 2 h high light stress (1200 µmol m^−2^ s^−1^).

### Arabidopsis chloroplast isolation and fractionation

Isolation and fractionation of intact chloroplasts was done as previously described (Hristou *et al.*, 2019; Stolle *et al.*, 2022, Preprint). Lysis of the chloroplasts was carried out at 1–2 mg chlorophyll ml^−1^ in HM buffer (10 mM magnesium chloride, 50 mM HEPES pH 8.0) for 30 min on ice, and thylakoids were separated from stroma by centrifugation (20 000 *g*, 4 °C, 10 min). Thylakoid samples were finally washed in HM buffer containing 150 mM sodium chloride and then adjusted to the desired chlorophyll concentration.

### Blue-native-polyacrylamide gel electrophoresis

Thylakoids isolated from chloroplasts of Arabidopsis plants (Col-0, *ffc*, cpSRP54/*ffc*, cpSRP54NG/*ffc*, cpSRP54∆C/*ffc*, cpSRP54(Q185R)/*ffc*) were solubilized with 1.5% (w/v) *n*-dodecyl-β-d-maltoside (DDM) at a chlorophyll concentration of 1 mg ml^−1^ for 20 min on ice. Following centrifugation (20 000 *g*, 4 °C, 10 min) blue native (BN)-PAGE samples were prepared using NativePAGE 5% G-250 sample additive (Thermo Fisher Scientific) at a final concentration of 0.25%. The multiprotein complexes were separated by a NativePAGE 4–16% BN-PAGE gradient according to the manufacturer’s instructions (Thermo Fisher Scientific) using total amounts of 10 µg chlorophyll/lane.

### Two-dimensional BN-PAGE/SDS-PAGE followed by immunoblot analysis

Multiprotein complexes of thylakoids from Arabidopsis plants (Col-0, *ffc*, cpSRP54∆C/*ffc*) were separated by BN-PAGE as described previously with adjusted application quantity according to the chlorophyll determination. Gel strips of the first dimensional NativePAGE 4–16% BN-PAGE gradient gel were incubated in preparation buffer (63 mM Tris pH 6.8, 33% (v/v) glycerol, 6.6% (w/v) SDS, 6.6% (v/v) β-mercaptoethanol, 6 M urea) for 1 h at 4 °C. For two-dimensional (2D) SDS-PAGE, multiprotein complexes were subsequently fractionated by electrophoresis on denaturing SDS gels (15% acrylamide) supplemented with 6 M urea (Schägger and Jagow, 1987). 2D gels were either stained with Coomassie Brilliant Blue R-250 or proteins were transferred onto polyvinylidene difluoride (PVDF) membranes by western blot. The dye was removed from the membrane by incubation in methanol. The membranes were subjected to immunoblot analysis with antibodies against PsbA, PsbD, PsaA, PsaB, PsaF, PsaK, Lhcb1, Lhca1, Lhca2, Lhca3, Lhca4, and PetA.

### Distribution determination of photosystem I core proteins in photosystem I* and photosystem I

ImageJ was used to determine protein levels of PsaA and PsaB on immunoblot band intensities after 2D SDS-PAGE. The measured values were calculated in relation to PSI* and PSI combined as 100%. Data were visualized in GraphPad Prism (v9.2.0).

### Sucrose density gradient centrifugation

Sucrose density gradient centrifugation was performed using stromal extracts of WT (Col-0), *ffc*, cpSRP54/*ffc*, cpSRP54NG/*ffc*, cpSRP54∆C/*ffc*, and cpSRP54(Q185R)/*ffc* corresponding to 500 µg chlorophyll before chloroplast fractionation. A loading sample (L) of stromal extract corresponding to 8 µg chlorophyll was taken and mixed with sample buffer. Sucrose layers of 10%, 20%, 30%, and 40% (w/v) sucrose in LSB buffer (20 mM HEPES, 50 mM potassium acetate, 6 mM magnesium acetate, 2 mM dithiothreitol, pH 7.5) were used for separation of compounds. After ultracentrifugation (175 000 *g*, 4 °C, 17 h) the gradients were fractionated. The sucrose concentration of each fraction was determined using a refractometer. Proteins contained in the fractions were precipitated in 10% trichloroacetic acid and the precipitates were analysed immunologically.

### Immunoblot analyses

Proteins separated by SDS-PAGE were blotted onto nitrocellulose or PVDF membranes. The immunological detection was performed using specific antibodies against PsbA (AS05 084), PsbD (AS06 146), PsbO (AS06 142-33), PsaA (AS06 172), PsaB (AS10 695), PsaF (AS06 104), PsaK (AS04 049), Lhcb1 (AS01 004), Lhcb4 (AS04 045), Lhca1 (AS01 005), Lhca2 (AS01 006), Lhca3 (AS01 007), Lhca4 (AS01 008), PetA (AS08 306), and uL4 (AS22 4787) obtained from Agrisera; His-tag (Penta-His horseradish peroxidase conjugate, ID 34460, Qiagen); Actin (A0480, Merck); cpSRP54M (Walter *et al.*, 2015a); and cpSRP43 (Klimyuk *et al.*, 1999).

### Total protein extracts of Arabidopsis leaves

Total protein extracts of 100 mg Arabidopsis leaves from 4-week-old plants (Col-0, *ffc*, cpSRP54/*ffc*, cpSRP54NG/*ffc*, cpSRP54∆C/*ffc*, cpSRP54(Q185R)/*ffc*) were prepared using TRIzol reagent (Thermo Fisher Scientific). Leaves were snap-frozen in liquid nitrogen and homogenized in 1 ml TRIzol reagent using a pistil. Samples were mixed with 200 µl chloroform and incubated for 10 min at RT followed by centrifugation (12 000 *g*, RT, 5 min). The pellets were resuspended in 300 μl 100% (v/v) ethanol, incubated for 3 min at RT, and again centrifuged (2000 *g*, 4 °C, 15 min). The resulting pellet was discarded, and the supernatant mixed with 1 ml isopropanol. After rotation-incubation for 30 min at RT and centrifugation (12 000 *g*, 4 °C, 10 min), the protein pellets were washed three times with 0.3 M guanidine hydrochloride in 95% (v/v) ethanol. A final washing step was carried out using 2 ml 100% (v/v) ethanol. Proteins were pelleted by centrifugation (7600 *g*, 4 °C, 5 min) and subsequently dried at 75 °C and 750 rpm. Protein pellets were resolved overnight in 100 µl 1% (w/v) SDS ultrapure followed by centrifugation (10 000 *g*, RT, 10 min) to get rid of insoluble compounds. The total protein extracts were mixed with sample buffer and subsequently applied to SDS-PAGE and western blot analyses. Samples were adjusted to Actin content for further immunoblot analyses. The protein levels of PsbA, PsbO, PsaA, PsaB, Lhcb1, Lhca2, and Actin were quantified based on immunoblot band intensities using ImageJ in relation to 100% wild type total protein extract.

### Statistical analyses

Statistical analyses and data visualization were performed in GraphPad Prism (v9.2.0). To determine statistically significant differences between means, one- or two-way analysis of variance (ANOVA) was followed by Šidák’s or Dunnett’s multiple comparisons test.

### Isothermal titration calorimetry

The thermodynamic values of recombinant cpSRP54 and cpSRP54(Q185R) binding to GDP and GTP were measured using a MicroCal ITC200 instrument (Malvern Panalytical). Protein and ligand concentrations were determined using a NanoDrop One spectrophotometer (Thermo Fisher Scientific). Protein at 25 µM and 1 mM ligand, diluted in SEC buffer (20 mM HEPES, pH 7.5, 200 mM NaCl, 20 mM MgCl_2_, and 20 mM KCl), were loaded in the cell and syringe, respectively. All measurements were done at 25 °C at a stirring rate of 750 rpm applying one injection of 0.4 µl and 12 more of 3 µl with a spacing of 150 s. Raw data were analysed in the MicroCal PEAQ-ITC Analysis Software v1.41 (Malvern Panalytical) applying the One Set of Sites model. The resulting thermodynamic parameters can be found in Supplementary Table S2.

### Determination of GTPase activity

The GTPase activity of recombinant cpSRP54 and cpSRP54(Q185R) in the presence of cpFtsY-NG was assayed in a buffer containing 25 mM HEPES (pH 7.5), 10 mM Mg(OAc)_2_, 300 mM K(OAc), 1 mM DTT, and 2.5% (v/v) glycerol. Enzymatic reactions containing 10 µM of each protein and 1 mM of GTP were incubated at 37 °C for up to 60 min. Samples taken at each corresponding time point were quenched by adding chloroform, vortexing, and subjecting them to 95 °C for 15 s, after which they were snap-frozen in liquid nitrogen. Thawed samples were centrifuged at 13 000 *g* for 5 min at 4 °C and the resulting aqueous phase was then analysed by HPLC in an Agilent 1260 Infinity system (Agilent) using a Metrosep A Supp 5–150/4.0 column (Metrohm). A flow rate of 0.6 ml min^−1^ in a buffer containing 90 mM (NH_4_)_2_CO_3_ (pH 9.25) was used and a wavelength of 260 nm was selected for nucleotide detection. GDP and GTP samples (Jena Bioscience) were used as standards to identify the retention time of these two nucleotides within the experimental samples. GTP consumption was determined by comparing the peak area corresponding to GTP at time=0 to the following time points.

### Protein expression and purification of recombinant proteins

Gene fragments encoding residues 65–366 of Arabidopsis cpFtsY and residues 79–564 of Arabidopsis cpSRP54 were cloned via GoldenGate (NEB) into a pET24d derivative (AL324, Novagen) modified for modular cloning for overproduction of C-terminal His6-tagged proteins (Kohm *et al.*, 2023). CpFtsY was cloned via *Bsa*I and cpSRP54 via *Esp*3I restriction sites resulting in AL324_cpFtsY-65 and AL324_cpSRP54 constructs. The overexpression constructs encoding mature cpFtsY and mature cpSRP54 described previously were used as templates (Bals *et al.*, 2010). The construct cpSRP54(Q185R) was generated using the QuikChange Lightning site-directed mutagenesis kit (Agilent Technologies) with pETDuet1-cpSRP54 (Bals *et al.*, 2010) serving as template. Primers are listed in Supplementary Table S1. The correct sequence of the constructs was verified by sequencing. His-tagged recombinant proteins were overexpressed in *Escherichia coli* strain Rosetta™ (DE3) and purified via an Äkta chromatography system using HisTrap columns (GE Healthcare, Waukesha, WI, USA). Washing (20 mM HEPES pH 8.0, 250 mM NaCl, 20 mM KCl, 40 mM imidazole) and elution (20 mM HEPES pH 8.0, 250 mM NaCl, 20 mM KCl, 250 mM imidazole) buffers did not contain MgCl_2_. Elution fractions were collected and supplemented with 30 mM EDTA for 20 min at RT. After concentration using centrifugal filters (Amicon Ultra, Witten, Germany), the elution was subjected to size exclusion chromatography (column XK16 S200, GE Healthcare) using a MgCl_2_-containing running buffer (20 mM HEPES pH 7.5, 200 mM NaCl, 20 mM MgCl_2_, 20 mM KCl). The elution fractions of the corresponding protein peak were pooled and concentrated as before, and the purified proteins were used for subsequent experiments.

### Size exclusion chromatography

Recombinant cpSRP54–His (Bals *et al.*, 2010) and cpSRP54(Q185R)–His (generated via site-directed mutagenesis as described in ‘Results’) were analysed using an ÄKTA-purifier system by size exclusion chromatography with a Superdex200 10/300 GL column (GE Healthcare) in 20 mM HEPES pH 7.5, 200 mM NaCl, 20 mM MgCl_2_, 20 mM KCl at a flow rate of 0.4 ml min^−1^. Selected fractions were then analysed by SDS-PAGE followed by Coomassie staining.

## Results

### Full complementation of the developmental defect of the *ffc* mutant requires expression of wild-type cpSRP54

To explore the function of the cpSRP54 protein *in vivo*, we generated four Arabidopsis complementation lines of the cpSRP54-knockout mutant *ffc1-2* (in the following referred to as *ffc*) (Amin *et al.*, 1999) using constructs encoding His-tagged cpSRP54 variants under the control of the 35S promoter. The complementation of the *ffc* mutant, which is reduced in growth and shows a chlorotic phenotype, particularly at a young developmental stage, was analysed by the expression of full-length cpSRP54 (cpSRP54/*ffc*), protein variants lacking the complete C-terminal half including the methionine-rich M-domain (cpSRP54NG/*ffc*) or its C-terminal tail region (cpSRP54∆C/*ffc*), and a point mutation variant, where a glutamine (Q) of the phosphate (P)-binding loop of the GTPase motif I (G1) in the G-domain was replaced by an arginine (R) (cpSRP54(Q185R)/*ffc*) (Fig. 1A). We selected this point mutation because previous studies demonstrated a detrimental effect of a mutation at this position on GTP hydrolysis in bacterial signal recognition particle (SRP) GTPases (Samuelsson *et al.*, 1995; Egea *et al.*, 2004; Shan *et al.*, 2004). After *Agrobacterium tumefaciens*-mediated transformation of the *ffc* plants, we screened the progeny of each transformation event, and lines with clearly measurable expression levels of the cpSRP54 variants were used for further analyses (Fig. 1B; Supplementary Fig. S1A, B). Furthermore, we confirmed the presence of the cpSRP54–His variants in stromal extracts of the plant lines by immunoblot analyses and verified their expected migration behavior by sucrose gradient centrifugation of the extracts (Supplementary Fig. S2).

The cpSRP54/*ffc* lines showed full complementation of the *ffc* mutant phenotype and exhibited similar rosette sizes and weights and even a slightly increased chlorophyll content compared with wild type plants (Fig. 1B–E). In contrast, the cpSRP54NG/*ffc* as well as the cpSRP54(Q185R)/*ffc* lines resembled the *ffc* mutant, showing significantly smaller and lighter plant rosettes than those of wild type (Fig. 1B–D). Comparable to the *ffc* mutant, the chlorophyll content was reduced to 56% and 54% of wild type level in cpSRP54NG/*ffc* and cpSRP54(Q185R)/*ffc*, respectively (Fig. 1B, E). The cpSRP54∆C/*ffc* plants, however, displayed a partial phenotypic complementation of the *ffc* mutant with more developed rosettes and a total chlorophyll content of 82% of wild type (Fig. 1B–E). Despite this, the overall phenotypic analysis of cpSRP54∆C/*ffc* revealed significant differences compared with the wild type plants (Fig. 1C–E).

### Impaired photosystem II activity of the *ffc* mutant is rescued by cpSRP54 lacking its C-terminal tail region, but not by cpSRP54 variants with a functionally impaired G-domain or lacking the M-domain

**Fig. 2.**
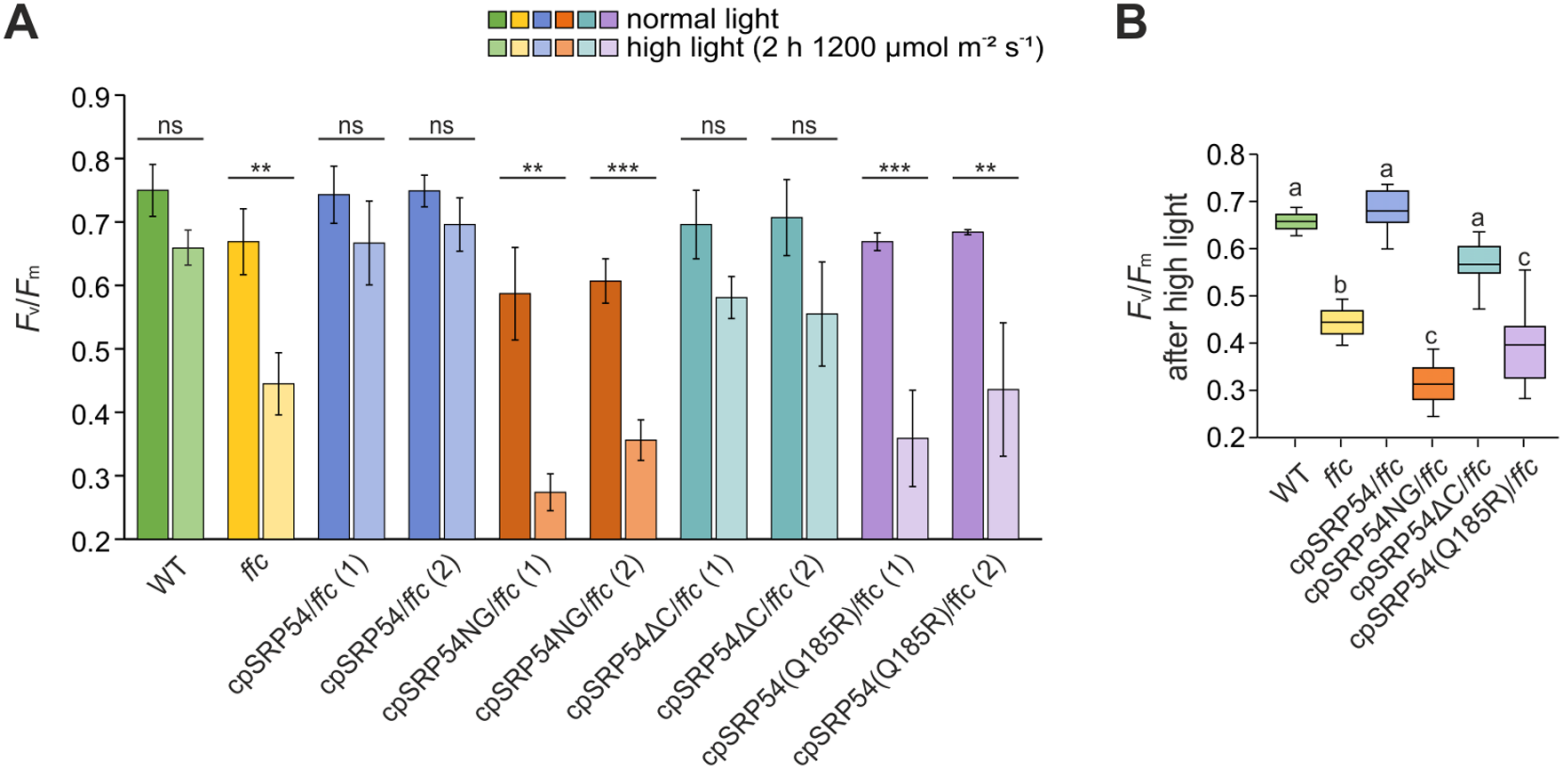
Measurement of the photosynthetic performance in Arabidopsis wild type, *ffc* and the *ffc*-complementation lines. The maximum quantum yield of photosystem II (PSII; *F*_v_/*F*_m_) of 8-week-old wild type (WT), *ffc*, and *ffc*-complementation plants was measured under constant normal light conditions and after 2 h high light stress. The *F*_v_/*F*_m_ values were calculated from three to five independent experiments with independently grown plants. (A) The means of normal and high light conditions were compared using multiple *t*-tests followed by the Holm–Šidák multiple comparisons method. Statistically significant differences are marked by asterisks (**P<0.01, ***P<0.001; ns, no significance, *P*>0.05). (B) The means of the measured *F*_v_/*F*_m_ after high light treatment were analysed using one-way ANOVA followed by Dunnett’s multiple comparisons test. Different letters indicate statistically significant differences (*P*<0.05) between means.

Since the *ffc* mutant shows a defect in PSII repair (Walter *et al.*, 2015b) we set out to determine the functionality of PSII in the complementation lines compared with wild type and *ffc.* Therefore, the maximum quantum efficiency of PSII in the dark-adapted state (*F*_v_/*F*_m_) was measured after the plants were exposed to normal (NL) and high (HL) light conditions. As expected, the cpSRP54/*ffc* plants showed *F*_v_/*F*_m_ values similar to wild type under both light conditions (Fig. 2A). In contrast to wild type and the fully complemented lines, *ffc* as well as cpSRP54NG/*ffc* and cpSRP54(Q185R)/*ffc* showed significant differences in the *F*_v_/*F*_m_ values between the NL and HL measurements indicating that high light treatment leads to a strong impairment of PSII activity in these plants (Fig. 2A). Here, the response of the cpSRP54NG/*ffc* and cpSRP54(Q185R)/*ffc* plants to high light was even more pronounced than that of *ffc* (Fig. 2B). This suggests that the expression of cpSRP54NG and cpSRP54(Q185R) disrupts the D1 repair cycle more severely than the complete absence of cpSRP54. This could be due to the unproductive binding of these variants to the cpSRP54 receptor, cpFtsY, which is likely involved in co-translational sorting in the *ffc* mutant (Tzvetkova-Chevolleau *et al.*, 2007; Asakura *et al.*, 2008; Walter *et al*, 2015b). Interestingly, expression of cpSRP54∆C in the *ffc* mutant complemented the impaired PSII activity of the *ffc* plants, and even under high light stress the cpSRP54∆C/*ffc* plants showed no significant difference in PSII activity compared with wild type (Fig. 2A, B). Together, the measurements suggest that the C-terminal tail region of cpSRP54 is not essential for biogenesis and maintenance of PSII, while the M-domain and a functional G-domain play important roles in these processes.

### Functional G- and M-domains of cpSRP54 are required for post- and co-translational cpSRP transport, while the C-terminal region is specific for post-translational sorting

**Fig. 3.**
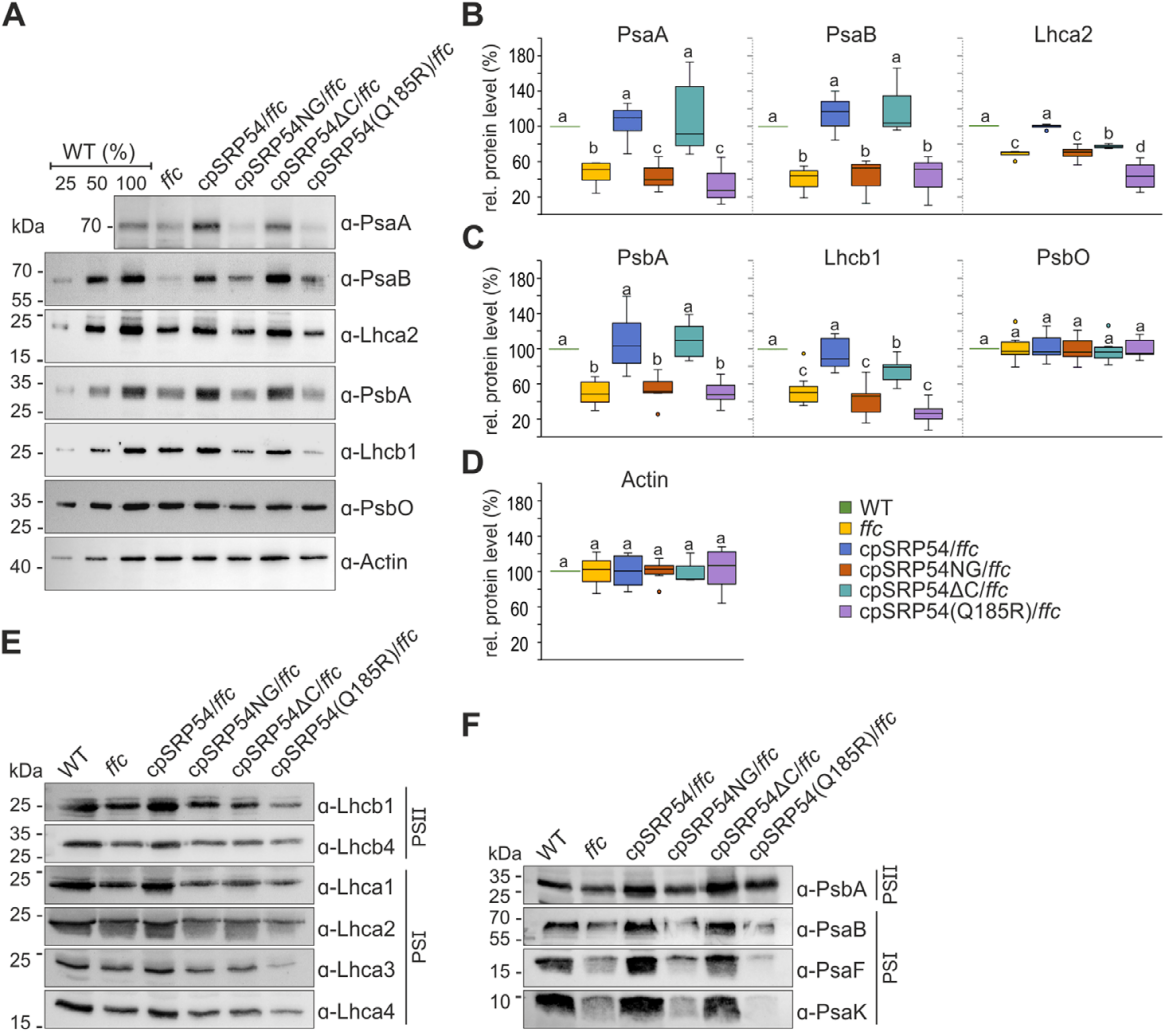
Immunoblot analysis of photosystem (PS) I and II subunits and light-harvesting chlorophyll *a*/*b*-binding proteins (LHCPs) in total protein extracts and thylakoid membranes of Arabidopsis wild type, *ffc*, and the *ffc*-complementation lines. (A) Total protein extracts from fresh leaf material of 4- to 5-week-old wild type (WT), *ffc*, and *ffc*-complementation plants were separated by SDS-PAGE and immunoblotted with the indicated antibodies. The WT extracts correspond to 25, 50, and 100% of total protein. The protein levels of PSI (B) and PSII (C) proteins, and the cytoplasmic protein Actin (D) were quantified by immunoblot band intensities using ImageJ in relation to wild type (100%). Three to eight biological replicates using fresh plant material were included in the analysis, depending on the antibody. Different letters indicate statistically significant differences (*P*<0.05) between means determined by one-way ANOVA followed by Dunnett’s multiple comparisons test. (E, F) Isolated thylakoids from 4- to 5-week-old WT and the transgenic lines were solubilized in *n*-dodecyl-β-d-maltoside and analysed by SDS-PAGE followed by immunoblot with the indicated antibodies against LHCPs (E) and photosystem subunits (F). One representative immunoblot of two experiments using thylakoids of independently grown plants is shown. The samples were separated based on the measured relative chlorophyll content of the plant lines (WT, 100%; *ffc*, 67%; cpSRP54/*ffc*, 124%; cpSRP54NG/*ffc*, 59%; cpSRP54∆C/*ffc*, 79%; cpSRP54(Q185R)/*ffc*, 54%). Uncropped images of the immunoblots are shown in Supplementary Figs S3, S4.

To determine the protein levels of PSI and PSII components in the thylakoid membrane of the complementation lines, *ffc*, and wild type, we performed an immunoblotting analysis using total protein extracts. This and all further experiments were carried out using one of the two complementation lines investigated so far. Here, we focused on the plastid-encoded PSI and PSII core subunits (PsaA, PsaB, and PsbA) and nuclear-encoded LHCPs (Lhca2 and Lhcb1), which are transported to the thylakoid membrane using the co- or post-translational cpSRP-dependent mechanism, respectively. The cytoplasmic protein Actin and the lumenal PSII subunit PsbO, which is transported via the cpSec1-dependent protein transport pathway, served as controls (Schünemann *et al.*, 1999). Quantification of the detected protein levels revealed that the observed protein levels in *ffc* (Fig. 3A) are in accordance with previous results (Amin *et al.*, 1999; Rutschow *et al.*, 2008; Hristou *et al.*, 2019), showing levels between 45% and 52% for PsaA, PsaB, and PsbA as well as 52% for Lhcb1 and 71% for Lhca2 compared with wild type (Fig. 3B, C).

The cpSRP54NG/*ffc* and cpSRP54(Q185R)/*ffc* lines showed no complementation of the PSI and PSII core subunits and the LHC proteins at all (Fig. 3B, C). A full recovery of the wild type protein level of all photosynthetic proteins was exclusively observed in the cpSRP54/*ffc* plants (Fig. 3A) exhibiting protein amounts of 104–117% of PsaA, PsaB, and PsbA, and 90–100% of Lhcb1 and Lhca2 compared with wild type (Fig. 3B, C). Interestingly, the cpSRP54∆C/*ffc* also showed pronounced complementation of the PSI and PSII reaction center proteins (92% PsaA, 104% PsaB and 109% PsbA of wild type level) but much less pronounced complementation in the level of Lhcb1 (75% of wild type level) and almost no complementation in the level of Lhca2 (77% of wild type level) (Fig. 3A–C). Since the use of total leaf protein extracts may involve the detection of proteins that are not integrated into the thylakoid membrane but accumulate in soluble fractions, immunoblotting experiments were performed using solubilized thylakoid membranes with chlorophyll concentrations based on the previously measured relative chlorophyll content of the plant lines (Fig. 2C). The analysed thylakoid membranes showed clear reductions in a variety of PSI and PSII LHCPs (PSI: Lhca1–4, PSII: Lhcb1, Lhcb4) in *ffc* and all complementation lines except cpSRP54/*ffc* (Fig. 3E). Moreover, while *ffc*, cpSRP54NG/*ffc*, and cpSRP54(Q185R)/*ffc* showed a reduction of the PSI and PSII reaction center subunits PsaB and PsbA, these subunits were markedly up-regulated in cpSRP54/*ffc* and cpSRP54∆C/*ffc*, displaying protein amounts comparable to wild type (Fig. 3F).

Taken together, the results demonstrate that the C-terminus of cpSRP54 plays an important role for the post-translational transport of LHCPs but is not required for co-translational transport of the PSI and PSII reaction center proteins *in vivo*. Furthermore, our data indicate that the M-domain and the GTPase activity of the G-domain are essential for the biogenesis of the PSI and PSII cores as well as for the LHCI and LHCII antenna systems.

### The loss of the cpSRP54 C-terminus promotes the accumulation of immature photosystem I

**Fig. 4.**
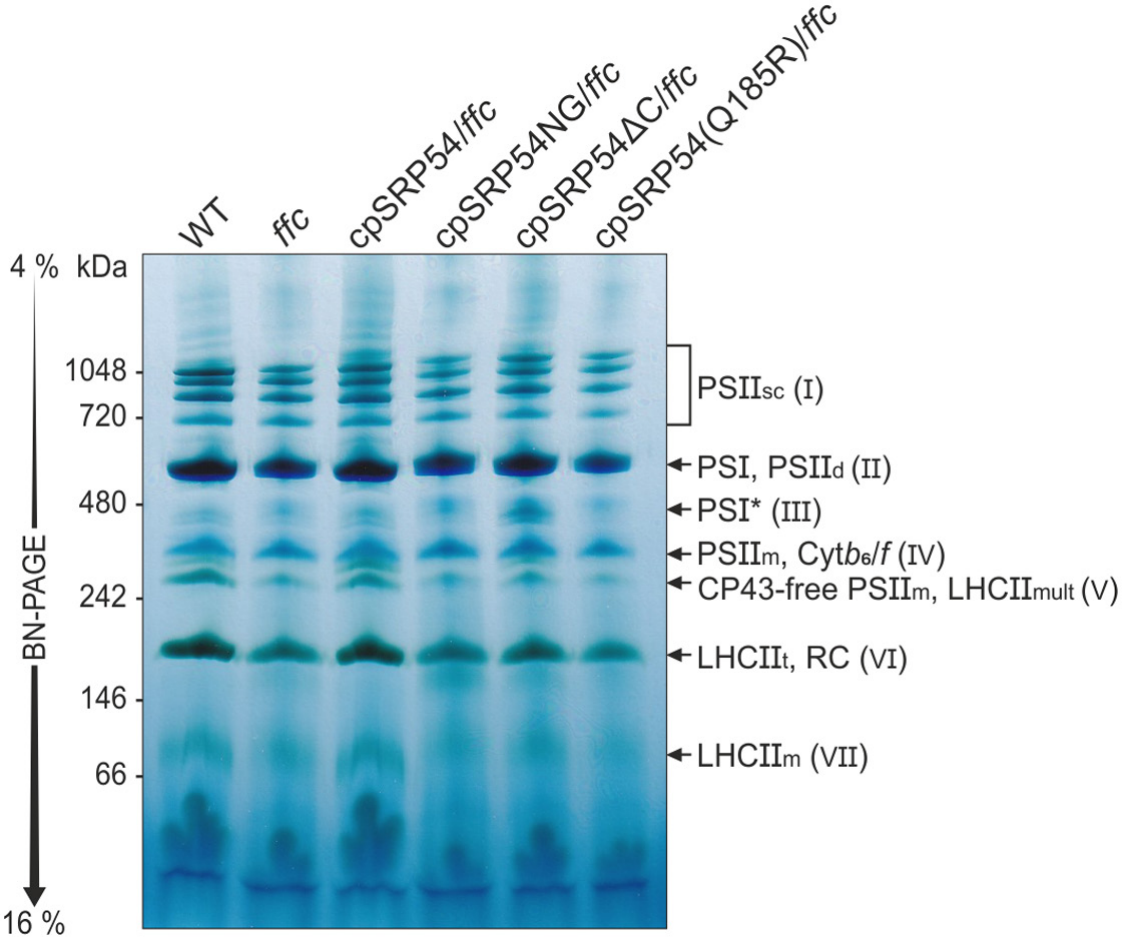
Blue native (BN)-PAGE analysis of thylakoid membrane multiprotein complexes of Arabidopsis wild type and the transgenic lines. Isolated thylakoids from 4- to 5-week-old wild type (WT), *ffc*, and *ffc*-complementation plants were solubilized in *n*-dodecyl-β-d-maltoside and separated by BN-PAGE based on their measured relative chlorophyll content (WT, 100%; *ffc*, 67%; cpSRP54/*ffc*, 124%; cpSRP54NG/*ffc*, 59%; cpSRP54∆C/*ffc*, 79%; cpSRP54(Q185R)/*ffc*, 54%). The identification of detected bands was accomplished in accordance with published BN-PAGE profiles of Arabidopsis thylakoids (Granvogl *et al.*, 2006; Armbruster *et al.*, 2010; Wittenberg *et al.*, 2017; Che *et al.*, 2022). CP43-free PSII_m_, V, CP43-free PSII monomers; Cyt*b*_*6*_/*f*, IV, cytochrome *b*_*6*_/*f* complex; LHCII_m_, VII, monomeric; LHCII_mult_, V, multimeric light harvesting antenna complex II; LHCII_t_, VI, trimeric LHCII; PSI and II, photosystem I and II; PSI*, III, PSI assembly intermediate; PSII_d_, II, PSII dimers; PSII_m_, IV, monomeric PSII; PSII_sc_, I, PSII supercomplexes; RC, VI, reaction center-like complex. Four independent BN-PAGE analyses using independently isolated thylakoids yielded similar results.

**Fig. 5.**
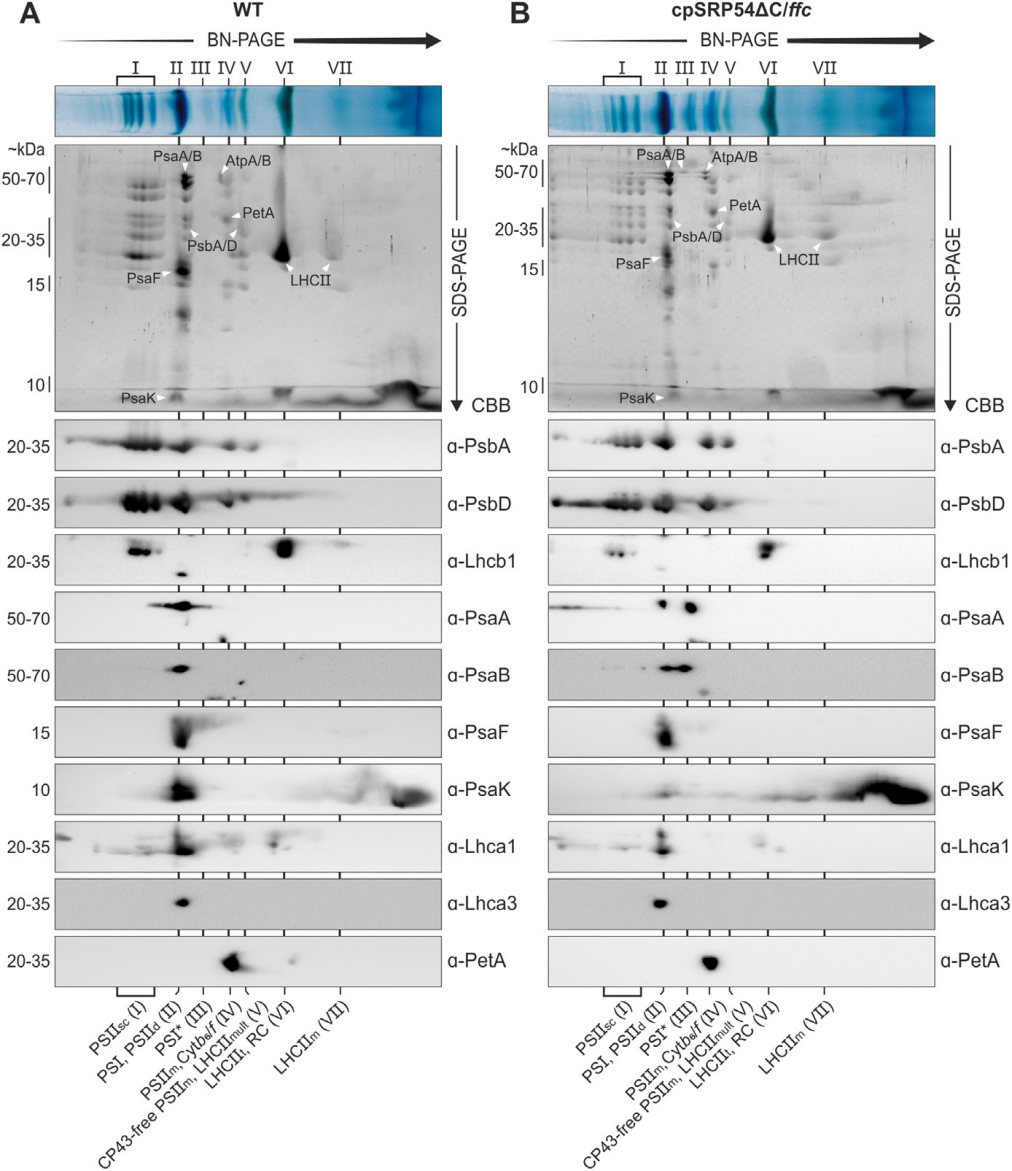
Two dimensional blue native (BN)-PAGE/SDS-PAGE analysis of thylakoid membrane multiprotein complexes in Arabidopsis wild type and the cpSRP54∆C/*ffc*-complementation line. Thylakoids of 4- to 5-week-old plants were solubilized in *n*-dodecyl-β-d-maltoside and separated by BN-PAGE based on their measured relative chlorophyll content. (A) Wild type (WT) 10 µg chlorophyll/lane; (B) cpSRP54C∆C/*ffc* 7.9 µg chlorophyll/lane. Detected bands were identified in accordance with published BN-PAGE profiles of Arabidopsis thylakoids (see legend to Fig. 4). The protein complex subunit composition was further determined by two-dimensional (2D) BN-PAGE/SDS-PAGE analysis followed by Coomassie brilliant blue (CBB) staining or western blot with subsequent immunodetection using the indicated antibodies. One representative result of two experiments using independently isolated thylakoids is shown. Uncropped images of the immunoblots are shown in Supplementary Fig. S5.

To obtain insight into the assembly of thylakoid membrane complexes in the cpSRP54 complementation lines, isolated thylakoids were treated with DDM, and the solubilized complexes subsequently fractionated by BN-PAGE. To analyse the relative abundances of the complexes in the complementation lines, samples were loaded according to the proportional chlorophyll concentrations of the plant lines with wild type serving as 100% control (Fig. 4). The complex distribution of wild type and cpSRP54/*ffc* showed similar patterns with four distinct PSII supercomplexes, fully assembled PSI and PSII dimers, monomeric PSII with the directly above-running Cyt*b*_*6*_*/f* complex, the CP43-free PSII monomer, the reaction center-like complex, as well as multimeric, trimeric, and monomeric LHCII at the expected sizes (Fig. 4). The same pattern of complexes, although in reduced amounts, was observed in *ffc* as well as in the complementation lines cpSRP54NG/*ffc*, cpSRP54(Q185R)/*ffc*, and cpSRP54∆C/*ffc*. Strikingly, however, cpSRP54∆C/*ffc* showed a clear accumulation of a complex (indicated as (III) in Fig. 4) with a running behavior similar to the previously described PSI* complex, representing an intermediate assembly state during PSI biogenesis in *Nicotiana tabacum* (Wittenberg *et al.*, 2017). To analyse the subunit composition of the accumulated complex in cpSRP54∆C/*ffc* and to identify further potential differences in the photosynthetic complexes of wild type and cpSRP54∆C/*ffc*, BN-PAGE slices were subsequently subjected to SDS-PAGE analysis (2D BN/SDS-PAGE) followed by Coomassie staining or immunoblot analyses (Fig. 5A, B). As the plastid-encoded Cyt*b*_*6*_/*f* complex subunit PetA is targeted independent from cpSRP54 by a pathway involving cpSecA1, it served as experimental control (Röhl and van Wijk, 2001). In cpSRP54∆C/*ffc* the PSII core subunits (PsbA and PsbD) were reduced in the PSII supercomplexes, while the levels of PSII dimeric and monomeric complexes and the CP43-free PSII assembly intermediate are similar to wild type. We also observed a reduction of Lhcb1 in the PSII supercomplexes in cpSRP54∆C/*ffc*. Furthermore, the level of Lhcb1 present as LHCII trimers was clearly reduced in this mutant. These findings are consistent with the data described above and corroborate that the cpSRP54∆C/*ffc* line is impaired in the biogenesis of LHCII, while the biogenesis of the PSII core is unaffected.

Notably, the PSI core subunits (PsaA and PsaB) displayed two clearly separated antibody signals in cpSRP54∆C/*ffc*, while only one signal was primarily detected in wild type (Fig. 5B). The signals could be assigned to the fully assembled PSI and the accumulated complex in cpSRP54∆C/*ffc* (Fig. 4).

Next, we wanted to test whether the subunits PsaF and PsaK, which were described to be absent or to occur only in low abundances in the PSI* complex of *N. tabacum* (Wittenberg *et al.*, 2017), are present in the PSI assembly intermediate. Both PSI subunits were reported to incorporate into the thylakoid membrane following the assembly of the PSI reaction center (Ozawa *et al.*, 2010; Wittenberg *et al.*, 2017; Zhang *et al.*, 2024). Immunodetection of PsaF and PsaK using solubilized thylakoid membranes showed no difference in the total amount of PsaF and PsaK between wild type and cpSRP54∆C/*ffc* (Fig. 3F). The 2D BN/SDS-PAGE immunoblot signals revealed that PsaF as well as PsaK is present in the fully assembled PSI but not in the PSI assembly intermediate. Remarkably, the amount of PsaK detected in the fully assembled PSI was drastically reduced in cpSRP54∆C/*ffc* while an accumulation as non-complexed protein was observed (Fig. 5B).

We also tested antibodies against LHCPs (Lhca1 and Lhca3) to determine whether the Arabidopsis PSI assembly intermediate is associated with antenna proteins, which are absent in the PSI* complex of *N. tabacum* (Wittenberg *et al.*, 2017). As shown in Fig. 5B, no co-migration of Lhca1 and Lhca3 with the PSI assembly intermediate was detected (Fig. 5B), while fully assembled PSI is associated with Lhcas to some extent. Notably, we were also able to detect small amounts of the PSI assembly intermediate in the *ffc* mutant (Supplementary Fig. S6). The observed antibody signals in the immunoblots after 2D BN/SDS-PAGE are consistent with the results of the analysis described above. Furthermore, immunoblots using antibodies against Lhca2 and Lhca4 clearly confirm that the Arabidopsis PSI assembly intermediate is not associated with LHCPs (Supplementary Fig. S6). In conclusion, the PSI assembly intermediate identified in Arabidopsis shares properties with the PSI* complex from *N. tabacum* and is henceforth referred to as PSI*.

**Fig. 6.**
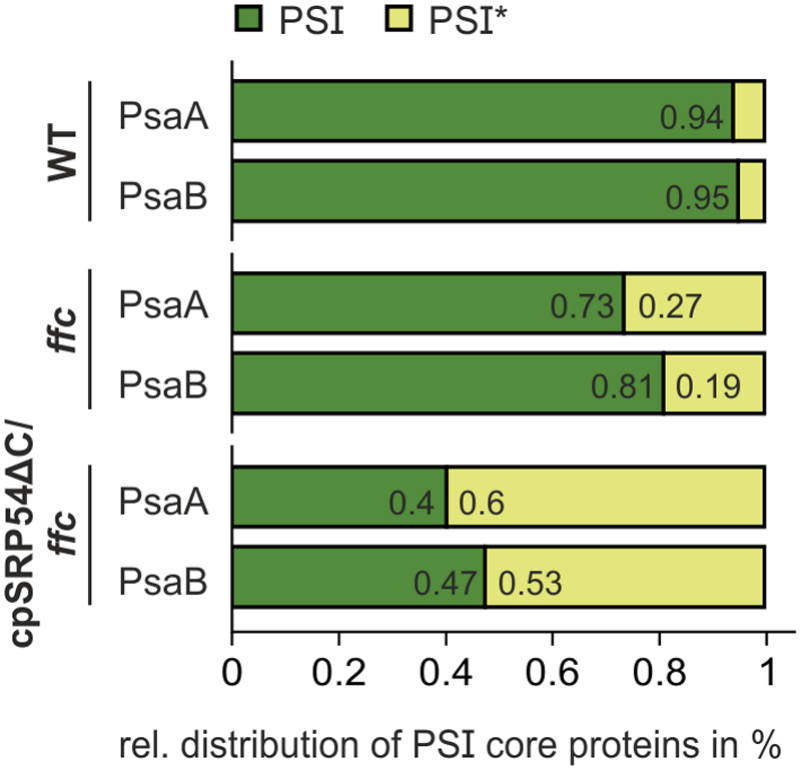
Distribution of the PSI core protein subunits. The protein levels of PsaA and PsaB in the PSI* assembly intermediate and the fully assembled PSI were determined by 2D blue native (BN)-PAGE/SDS-PAGE immunoblot band intensities using ImageJ. Data derived from two (WT and *ffc*) and three (cpSRP54C∆C/*ffc*) independent experiments using independent plant material. The total immunoblot signal calculated from both PSI* and PSI served as 100%.

To analyse the relative abundance of the PSI and PSI* complexes in wild type, *ffc*, and cpSRP54∆C/*ffc*, the signals of the PSI core subunits in the immunoblots were quantified. While in wild type the vast majority were assigned to the fully assembled PSI (PSI: 94% PsaA, 95% PsaB), the distribution in *ffc* was altered (PSI: 73% PsaA, 81% PsaB; PSI*: 27% PsaA, 19% PsaB) and in cpSRP54∆C/*ffc* even markedly shifted towards the PSI* complex (PSI: 40% PsaA, 47% PsaB; PSI*: 60% PsaA, 53% PsaB) (Fig. 6).

Taken together, our data show that the impaired biogenesis of LHC proteins combined with the ongoing co-translational formation of the PSI core, as seen in the cpSRP54∆C/*ffc* line, leads to a strong accumulation of PSI*. Furthermore, our data indicate that stable association of PsaK in PSI is dependent on the formation of the LHC antenna.

### Point mutation in the P-loop of cpSRP54 decelerates its GTPase activity

**Fig. 7.**
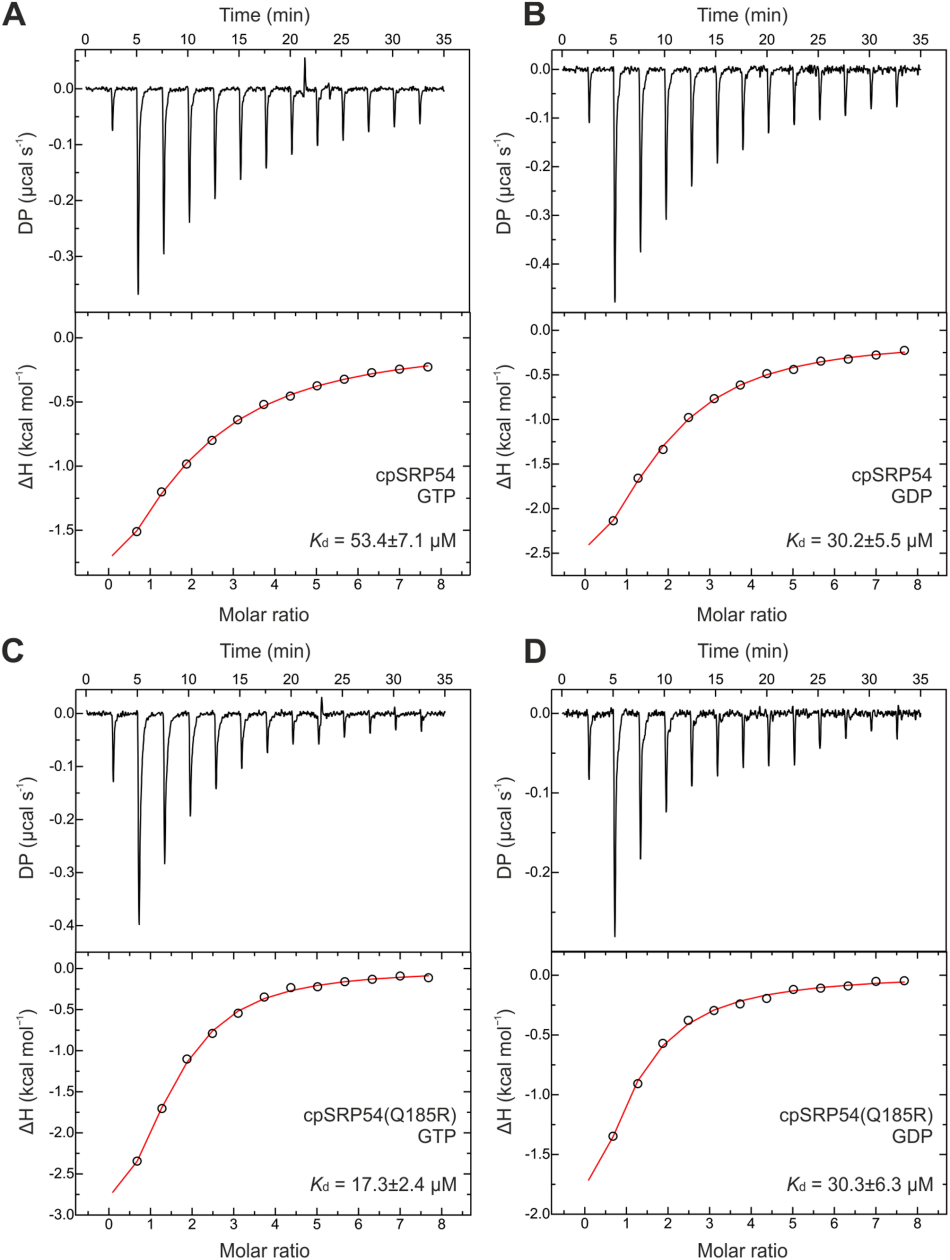
Isothermal titration calorimetry measurement to determine binding affinities for the cpSRP54 protein and the point mutant cpSRP54(Q185R) with GTP and GDP. One millimolar of the ligand (GTP or GDP) was titrated with 25 µM of cpSRP54–His or cpSRP54(Q185R)–His. The resulting variations in heating power were recorded and the enthalpy changes were plotted versus the molar ratio of cpSRP54 or cpSRP54(Q185R) and the corresponding nucleotides. The titration isotherms yielded the average *K*_d_ values and SDs from triplicate runs with the same protein batch indicated. (A) cpSRP54/GTP; (B) cpSRP54/GDP; (C) cpSRP54(Q185R)/GTP; (D) cpSRP54(Q185R)/GDP. The thermodynamic parameters from each replicate are shown in Supplementary Table S2.

As our data showed that the cpSRP54(Q185R)/*ffc* plants exhibit a drastic impairment in plant development and significantly reduced levels of photosynthetic proteins and high molecular mass protein complexes, the behavior of cpSRP54(Q185R) was further investigated *in vitro*. Therefore, we quantitatively analysed the interaction of recombinant cpSRP54–His and cpSRP54(Q185R)–His with either GTP or GDP using isothermal titration calorimetry. The measurements using cpSRP54–His resulted in an average dissociation constant of 53.4 µM (±7.1) with GTP (Fig. 7A), and 30.2 µM (±5.5) with GDP (Fig. 7B). For the titration of cpSRP54(Q185R)–His an average dissociation constant of 17.3 µM (±2.4) with GTP (Fig. 7C) and 30.3 µM (±6.3) with GDP (Fig. 7D) was determined. Despite the affinity of GDP being the same for both proteins, the affinity of GTP for the point-mutated protein cpSRP54(Q185R)–His was ~3-fold higher than for the wild type protein cpSRP54–His.

**Fig. 8:**
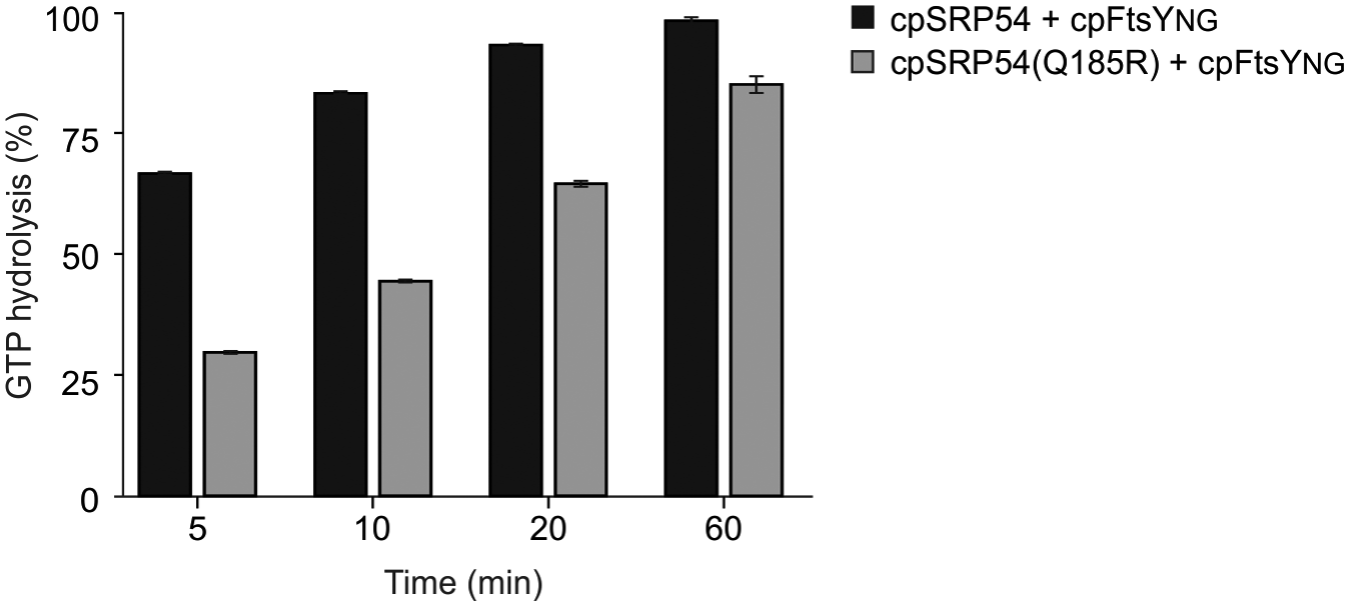
GTPase activity of cpSRP54 and cpSRP54(Q185R). The GTPase activity of cpSRP54–His and cpSRP54(Q185R)–His was measured by HPLC in the presence of cpFtsYNG–His using 10 µM of each protein and 1 mM of GTP. Data are the mean values and SDs of three independent enzymatic reactions with the same protein batch. The GTP hydrolysis was measured at different time points up to 60 min, and the consumption was determined in relation to the 0 min time point.

Moreover, we determined the GTPase activity of wild type and the point mutated proteins using HPLC. As cpSRP54 and its receptor protein cpFtsY were described as stimulating their mutual GTPase activity through interaction of their homologous G-domains (Jaru-Ampornpan *et al.*, 2009; Wild *et al.*, 2016), the experiments were performed in the presence of cpFtsYNG–His. The GTP consumption was determined at different time points and set in relation to the initial GTP amount. After 5, 10, and 20 min the cpSRP54-containing samples exhibited a GTP consumption of 67%, 83%, and 93%, respectively. After 60 min the GTP was hydrolysed to almost 100% (Fig. 8). In comparison, GTP consumption was greatly reduced in the cpSRP54(Q185R)-containing samples. This was most prominent after relatively short incubation times of 5, 10, and 20 min, which resulted in the hydrolysis of 30%, 44%, and 65% GTP (Fig. 8). Overall, our data indicate that the higher affinity of cpSRP54(Q185R) to GTP traps the protein more persistently in its GTP-bound state, which results in a reduced GTP hydrolysis rate.

To exclude the possibility that the point-mutation causes misfolding of the protein, size exclusion chromatography was performed, which demonstrated the same running behavior for cpSRP54–His and cpSRP54(Q185R)–His (Supplementary Fig. S7).

## Discussion

The role of the cpSRP54 protein within the SRP-dependent protein transport of higher plant chloroplasts has been characterized in several studies in the last decades. However, most studies were restricted to *in vitro* experiments, and questions regarding the precise role of cpSRP54 *in vivo* remained open. Our study revealed a deeper insight into the functional role of the individual domains of cpSRP54 *in vivo.* The domains are similarly organized as in the homologous SRP54 proteins of eukaryotes and prokaryotes (Ffh), which mediate the co-translational protein transport to the endoplasmic reticulum and the plasma membrane, respectively (Franklin and Hoffman, 1993; Grudnik *et al.*, 2009; Akopian *et al.*, 2013). However, cpSRP54 differs from its cytosolic homologues by a land plant-specific C-terminal tail region harboring the positively charged cpSRP43-binding motif (ARRKR in Arabidopsis) (Funke *et al.*, 2005). This motif is essential for the formation of the high affinity cpSRP54–cpSRP43 heterodimeric complex in Arabidopsis (Ziehe *et al.*, 2018). Furthermore, *in vitro* experiments to reconstitute transit complex formation with LHCP and insertion of LHCP into thylakoid membranes indicated that complex formation between the cpSRP subunits is essential for these processes (Schünemann *et al.*, 1998; Tu *et al.*, 1999; Yuan *et al.*, 2002; Goforth *et al.*, 2004; Funke *et al.*, 2005). However, more recent *in vitro* studies suggested that cpSRP43 alone might be sufficient for LHCP targeting because it was demonstrated that cpSRP43 prevents LHCP aggregation by itself (Falk and Sinning, 2010; Jaru-Ampornpan *et al.*, 2010) and is able to directly contact the Alb3 translocase in the thylakoid membrane (Bals *et al.*, 2010; Falk *et al.*, 2010; Lewis *et al.*, 2010; Dünschede *et al.*, 2011). A cpSRP43-mediated LHCP sorting was also indicated by the analysis of cpSRP-pathway Arabidopsis mutants. Notably, however, this pathway was only up-regulated in the absence of cpSRP54 as well as its thylakoid receptor cpFtsY (Tzvetkova-Chevolleau *et al.*, 2007). In this study, we show that a cpSRP54 variant that lacks the C-terminal tail region is not able to rescue the defect in post-translational LHCP transport in the *ffc* mutant. Therefore, our results are consistent with the *in vitro* LHCP insertion experiments and support the view that efficient cpSRP-dependent LHCP targeting to the thylakoid membrane depends on complex formation between cpSRP43 and cpSRP54 *in vivo*. Since our data suggest that free cpSRP54 does not support LHCP insertion, it will be interesting to analyse the transport of LHCP proteins in the cpSRP43-knockout mutant *chaos*, which shows reduced but still considerable amounts of LHCPs (Amin *et al.*, 1999; Klimyuk *et al.*, 1999).

Our findings substantiate the hypothesis that the evolution of the C-terminal tail region was likely triggered to facilitate efficient post-translational sorting of the LHCP proteins in land plants (Dünschede *et al.*, 2015). Consistent with this, our data indicate that the plant-specific C-terminal region of cpSRP54 is dispensable for the co-translational transport of the photosynthetic reaction center subunits, while the cpSRP54 G and M domain, which are conserved in all SRP systems, are important for co-translational sorting.

Recent *in vitro* data demonstrated that the C-terminal tail region and a binding motif within the M domain contribute, along with the NG domain, to the interaction of cpSRP54 with ribosomes and are critically involved in forming the contact between cpSRP54 and the ribosomal subunit uL4 (Hristou *et al.*, 2019). It has been suggested that the cpSRP54–uL4 contact may initiate co-translational sorting at a very early stage of translation (Hristou *et al.*, 2019). Our study shows that the deletion of the C-terminal region of cpSRP54 does not impair co-translational sorting, questioning the physiological relevance of the cpSRP54–uL4 contact. However, it is possible that preventing cpSRP54–uL4 binding *in vivo* requires the removal of both uL4 binding domains located in the C-terminal region and the M-domain. In line with this assumption is our observation that the cpSRP54ΔC variant exhibits some co-fractionation with ribosomes (Supplementary Fig. S2). Nonetheless, the molecular details of this interaction remain unclear and might involve other contact sites between cpSRP54 and the ribosome.

Interestingly, the function of the M domain in co-translational sorting in chloroplasts can be attributed to an important role in the docking of the ribosome nascent chain complex to the thylakoid membrane as it greatly accelerates the binding to its receptor, cpFtsY, a function that is mediated by the SRP RNA in the bacterial system (Jaru-Ampornpan *et al.*, 2009). By analogy to the bacterial SRP system, it can be speculated that the M domain is additionally able to bind directly to the nascent chain, while it emerges out of the ribosomal peptide tunnel exit site. A direct contact between cpSRP54 and the nascent chain of D1 has already been demonstrated, but molecular details of this interaction are currently unclear (Nilsson *et al.*, 1999; Nilsson and van Wijk, 2002).

The complex formation of the SRP GTPases cpSRP54–cpFtsY is GTP-dependent as binding of the nucleotide leads to conformational changes of the proteins enabling dimerization and therefore reciprocal GTPase activation (Jaru-Ampornpan *et al.*, 2007; Nguyen *et al.*, 2011; Wild *et al.*, 2016). While *in vitro* experiments demonstrated that LHCP insertion into thylakoid membranes is indeed GTP-dependent (Hoffman and Franklin, 1994; Yuan *et al.*, 2002), the importance of GTP hydrolysis for co-translational targeting in chloroplasts remained unknown. Here, we observed that a point mutation within the highly conserved GI motif of cpSRP54 at amino acid position 185 dramatically affects the functionality of cpSRP54 in post- and co-translational sorting of LHCPs and plastid-encoded photosynthetic reaction center proteins, respectively, *in vivo*. The GI motif, also known as the Walker-A motif or P-loop, is part of the active center for GTP binding/hydrolysis and interacts with the β-phosphate of GTP by forming an anion hole (Shan, 2016; Wild *et al.*, 2016). Therefore, it seems reasonable that mutations within the GI motif can affect the nucleotide affinity and GTP hydrolysis rate as we observed for the cpSRP54(Q185R) variant.

Our investigation of thylakoid membrane complexes revealed that an intermediate assembly state of PSI (PSI*) is slightly up-regulated in *ffc* and greatly accumulates in cpSRP54∆C/*ffc*. This complex is composed of a subset of PSI core proteins and lacks the LHCI antenna proteins. Therefore, it resembles previously described PSI assembly intermediates in *N. tabacum*, *Chlamydomonas reinhardtii*, and a recently identified rice mutant lacking cpSRP54 (Ozawa *et al.*, 2010; Wittenberg *et al.*, 2017; Gao *et al.*, 2022). The specific accumulation of this assembly complex in the *ffc* mutant expressing cpSRP54 lacking its C-terminal region can be explained by our findings that this cpSRP54 variant still supports the co-translational biogenesis of the PSI core proteins PsaA and PsaB, while the post-translational transport of the LHCI antenna proteins is impaired. Currently it is known that PSI assembly in land plants begins with the formation of the PsaA–PsaB heterodimer and continues with the integration of a specific subset of peripheral subunits (PsaC–E, PsaH, PsaI, and PsaL) to form PSI*, a stable PSI core assembly intermediate. Subsequently, the addition of PsaF is required to trigger full assembly of PSI by the integration of five additional subunits, including PsaK, and the LHCI proteins (Schöttler *et al.*, 2011; Wittenberg *et al.*, 2017; Zhang *et al.*, 2024). Notably, we observed that the reduced assembly of LHCI to PSI leads to a reduced amount of PsaK in PSI. In this respect, it is important to note that PsaK was shown to integrate into the thylakoid membrane independently of cpSRP54 (Mant *et al.*, 2001). Furthermore, the cpSRP54∆C/*ffc* plant shows a specific down-regulation of PsaK in PSI but no changes in the total amount of PsaK demonstrating that PsaK insertion itself is not impaired in cpSRP54∆C/*ffc*. Therefore, our data indicate that the stable integration of PsaK in PSI needs the assembly of LHCI and likely presents one of the last steps in PSI maturation in land plants.

## Supplementary data

The following supplementary data are available at *JXB* online.

Fig. S1. Identification of Arabidopsis *ffc*-complementation lines.

Fig. S2. Sucrose density gradient analysis of stromal extracts from Arabidopsis wild type (WT), *ffc*, and *ffc*-complementation lines.

Fig. S3. Uncropped images of the immunoblots shown in Fig. 3A.

Fig. S4. Uncropped images of the immunoblots shown in Fig. 3E, F.

Fig. S5. Uncropped images of the immunoblots shown in Fig. 5.

Fig. S6. Two-dimensional BN-PAGE/SDS-PAGE and immunoblot analysis of thylakoid membrane multiprotein complexes in Arabidopsis *ffc.*

Fig. S7. Analysis of the migration behavior of recombinant cpSRP54 and the point mutation variant cpSRP54(Q185R).

Table S1. Primers used for generation of transformation constructs, site-directed mutagenesis, and genotyping PCRs.

Table S2. Isothermal titration calorimetry (ITC) parameters—thermodynamic values determined from the ITC experiments.

erae293_suppl_Supplementary_Tables_S1-S2_Figures_S1-S7

## Data Availability

All relevant data are provided in the article and its supplementary data.

## Abbreviations:

cpSRP: , chloroplast signal recognition particle
LHCI: light-harvesting antenna complexes I
LHCII: light-harvesting antenna complexes II
LHCPs: light-harvesting chlorophyll *a*/*b*-binding proteins
PSI: photosystem I
PSII: photosystem II;
SRP: signal recognition particle
