# Supplementary material for: The role of chloroplast SRP54 domains and its C-terminal tail region in post- and co-translational protein transport in vivo
Source: J Exp Bot. 2024 Jul 11;75(18):5734–49. doi: 10.1093/jxb/erae293 (PMC11427828; doi:10.1093/jxb/erae293)
Supplement: erae293_suppl_Supplementary_Tables_S1-S2_Figures_S1-S7 [file erae293_suppl_supplementary_tables_s1-s2_figures_s1-s7.pdf]

Supplementary Data – Bischoff *et al*.

Supplementary Figures

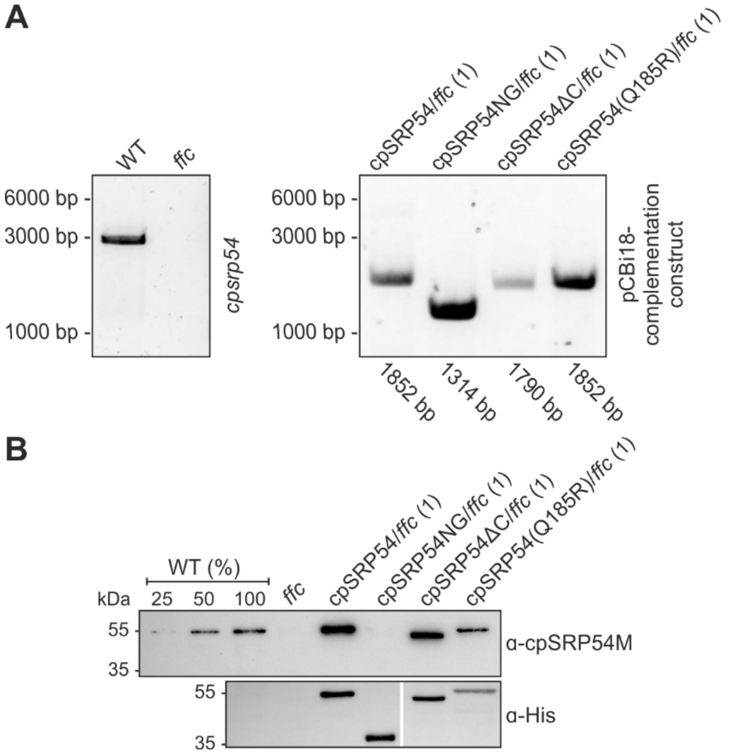

Supplementary Figure S1: Identification of *A. thaliana ffc*-complementation lines.
The *ffc*-complementation lines were generated by *Agrobacterium tumefaciens*-mediated plant transformation. The different cpSRP54 variants were transformed in the cpSRP54-lacking mutant (*ffc*) background. (**A**) Putative *ffc*-complementation lines were analyzed by genotyping PCRs. Plants of the wild type Columbia-0 (WT) and *ffc* served as controls. Specific primers for gene identification of *cpsrp54* (At5g03940) or the pCBi18-complementation construct were used to identify correct transformation lines (Supplementary Table S1). One representative *ffc*-complementation line is exemplarily shown for each cpSRP54 variant. (**B**) Total protein extracts from fresh leaf material of 4- to 5-week-old wild type (WT), *ffc* and *ffc*-complementation plants were separated by SDS-PAGE and immunoblotted with antibodies against cpSRP54M and the His-tag. The WT extracts correspond to 25%, 50%, and 100% of total protein.

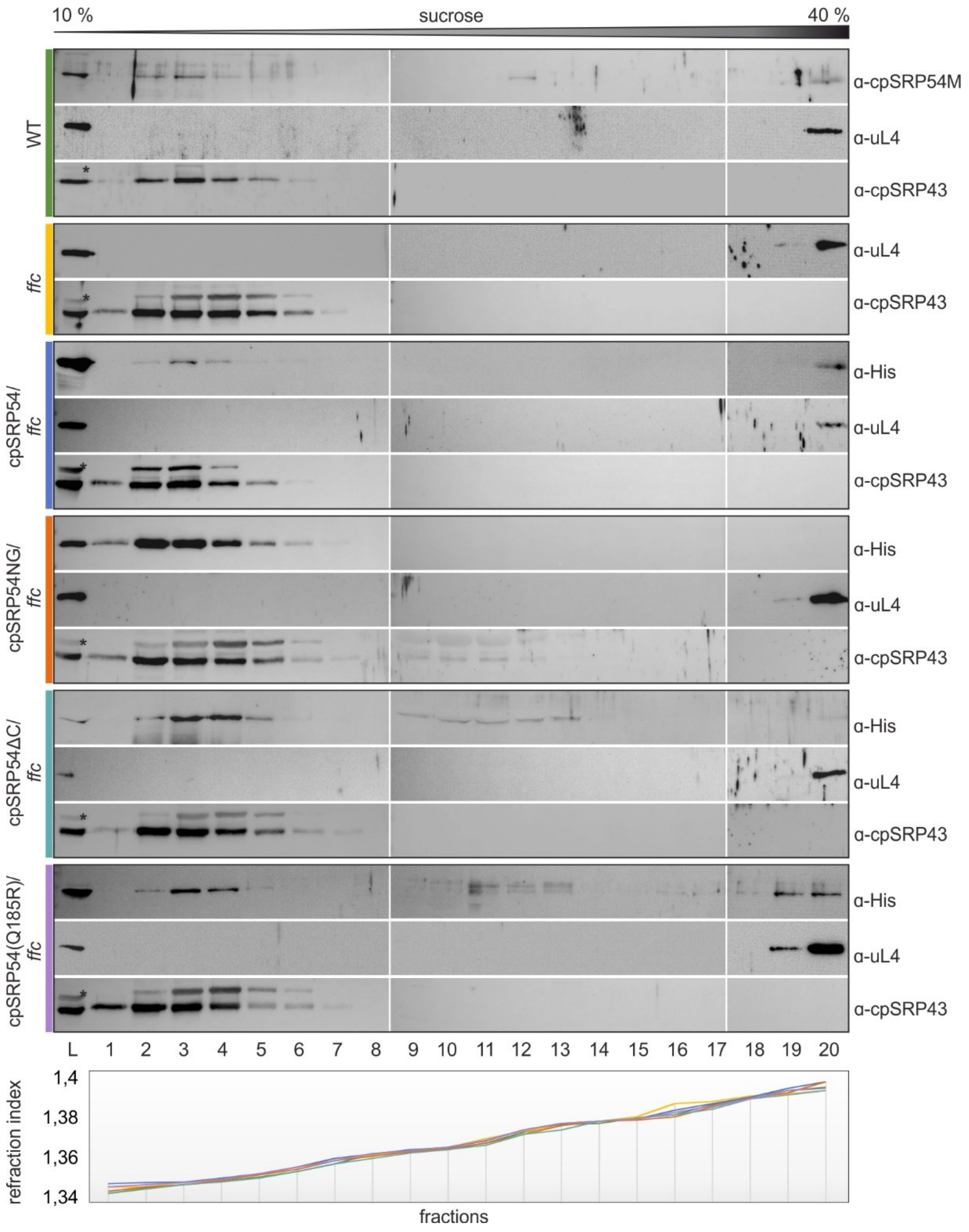

Supplementary Figure S2: Sucrose density gradient analysis of stromal extracts from *A. thaliana* wild type (WT), *ffc* and *ffc*-complementation lines.

The stromal extracts of fractionated chloroplasts were loaded onto a 10-40% sucrose gradient at a concentration of 500 µg chlorophyll before chloroplast fractionation. After ultracentrifugation, the sucrose concentration of the gradient fractions was determined using a refractometer. The fractions were protein precipitated using trichloroacetic acid and subsequently analyzed by immunoblot with antibodies against cpSRP54M, cpSRP43, His-tag, and the chloroplast ribosomal subunit uL4. The asterisk (*) marks a nonspecific cross-reaction of the cpSRP43 antibody. A loading sample (L) of stromal extract served as experimental control. As expected, the wild type cpSRP54 occurred in two pools. A clear co-sedimentation with the chloroplast ribosomes was detected for wild-type cpSRP54, cpSRP54-His, and cpSRP(Q185R)-His. CpSRP54ΔC-His shows a weak association with ribosomes, while cpSRP54NG-His was not detected in the ribosomal fractions. In all plant lines the respective cpSRP54 variant comigrated with cpSRP43 in the fractions with lower sucrose (<20%) concentrations, which either contained the cpSRP54/cpSRP43 complex or free cpSRP54.

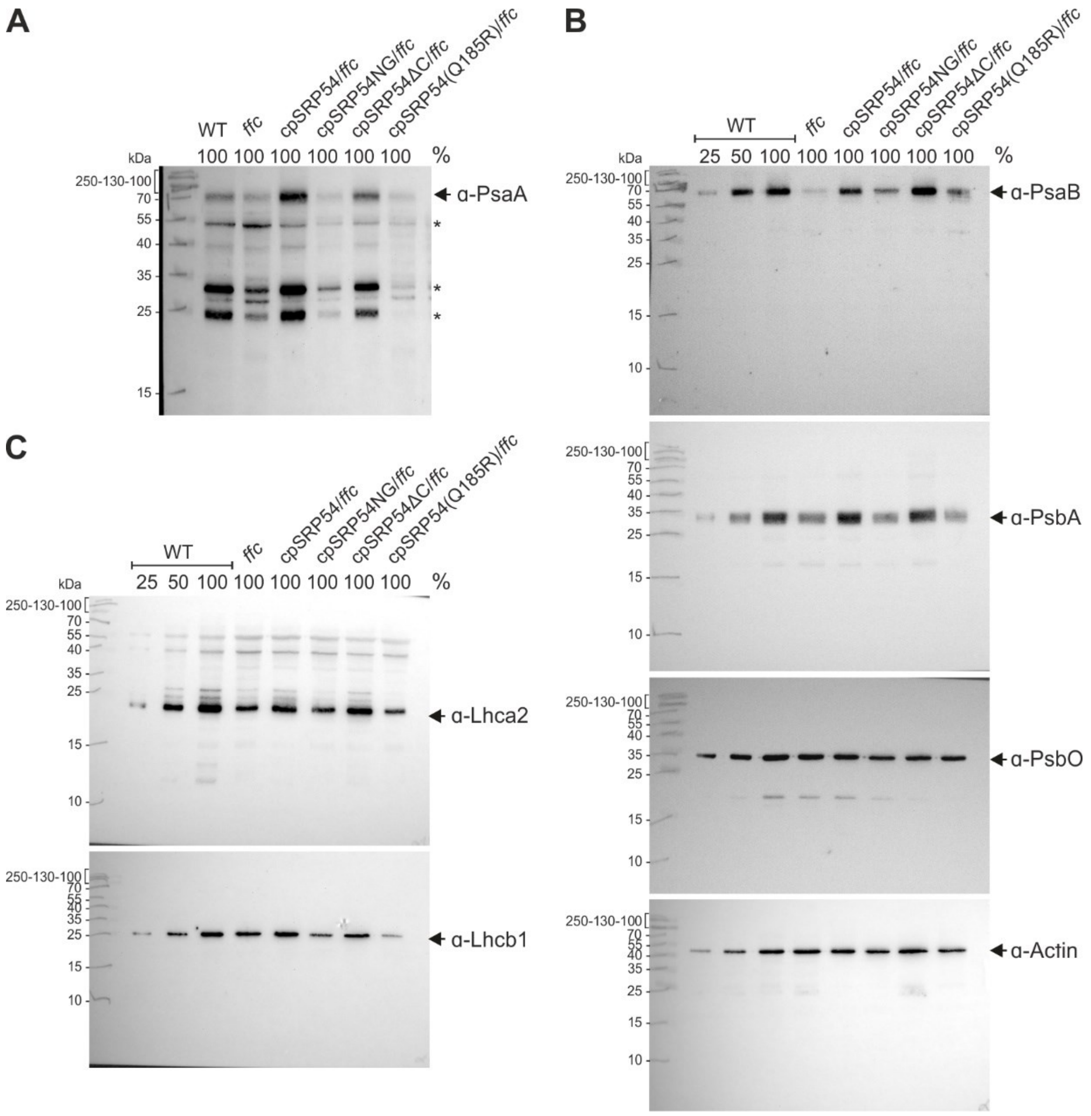

Supplementary Figure S3: Uncropped images of the immunoblots shown in Figure 3A.
Uncropped immunoblots as shown in Figure 3A depicting an analysis of total protein extracts of *A. thaliana* wild type, *ffc* and the *ffc*-complementation lines using antibodies against PSI and PSII subunits and LHCPs. (**A**) Uncropped immunoblot using an α-PsaA antibody. The asterisks (*) mark cross-reactions of the PsaA antibody, which were observed multiple times in experiments with extracts from fresh leaf material. (**B**) Uncropped immunoblots using antibodies against PsaB, PsbA, PsbO, Actin, and (**C**) Lhca2 and Lhcb1. The α-Actin antibody was used as loading control to adjust the samples to 100% total protein extract. For better visualisation the protein ladder was additionally marked with a pencil line on the blots (see also Supplementary Figures S6 and S7).

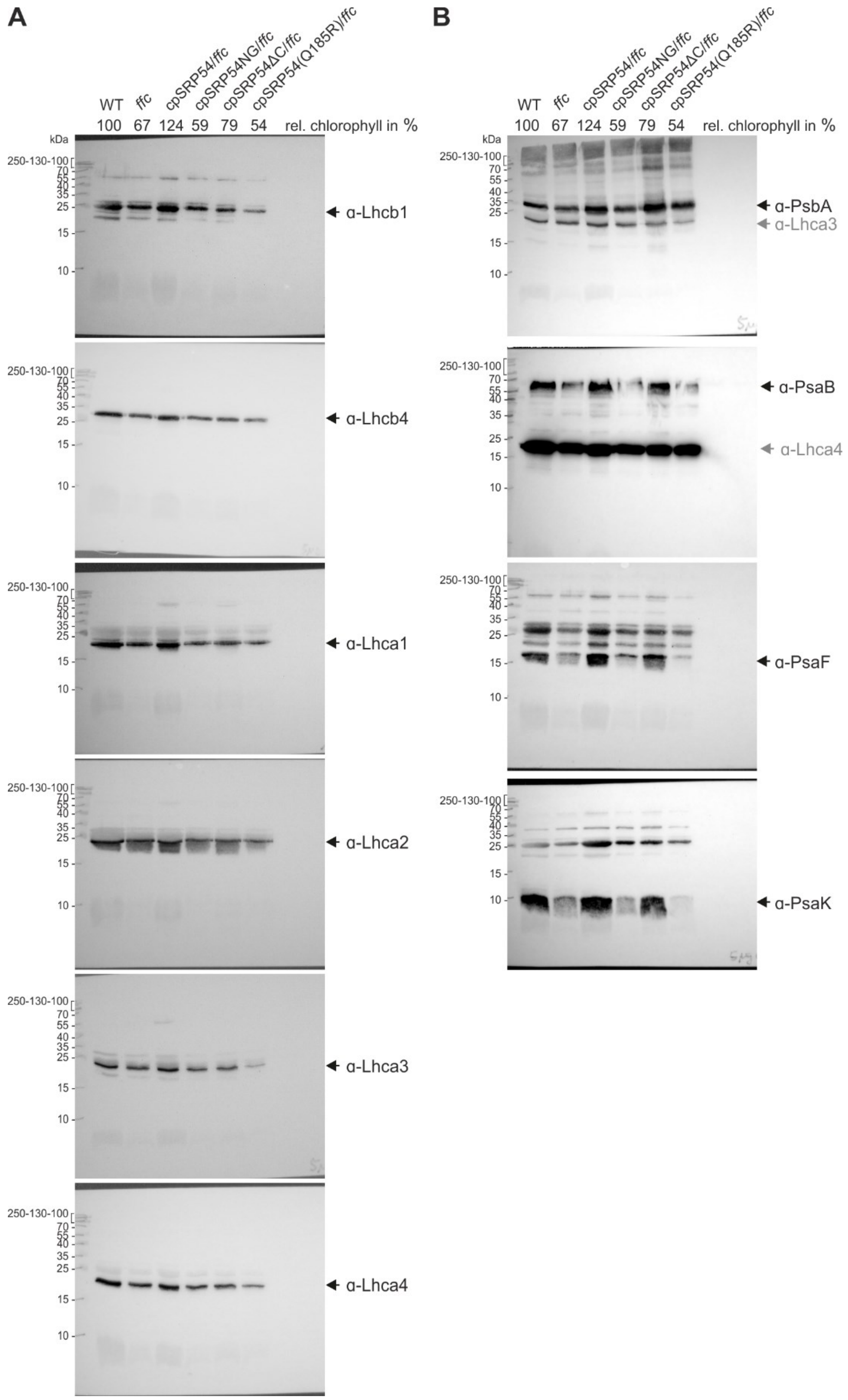

Supplementary Figure S4: Uncropped images of the immunoblots shown in Figure 3E/F. Uncropped immunoblots as shown in Figure 3E/F depicting an analysis of solubilized thylakoids of the *A. thaliana* wild type, *ffc* and the *ffc*-complementation lines. The sample loading was adjusted according to the measured chlorophyll content of the different plant lines as shown in Figure 1E. Immunoblots are labelled with antibodies against (**A**) LHCPs (α-Lhcb1, α-Lhcb4, α-Lhca1-4) and (**B**) PSI and PSII subunits (α-PsbA, α-PsaB, α-PsaF, α-PsaK). Antibody signals labelled in black were used to generate the cropped figures in the main text. Antibodies labelled in grey were previously detected on the same blot membrane. The membranes were then used for further immunodetection.

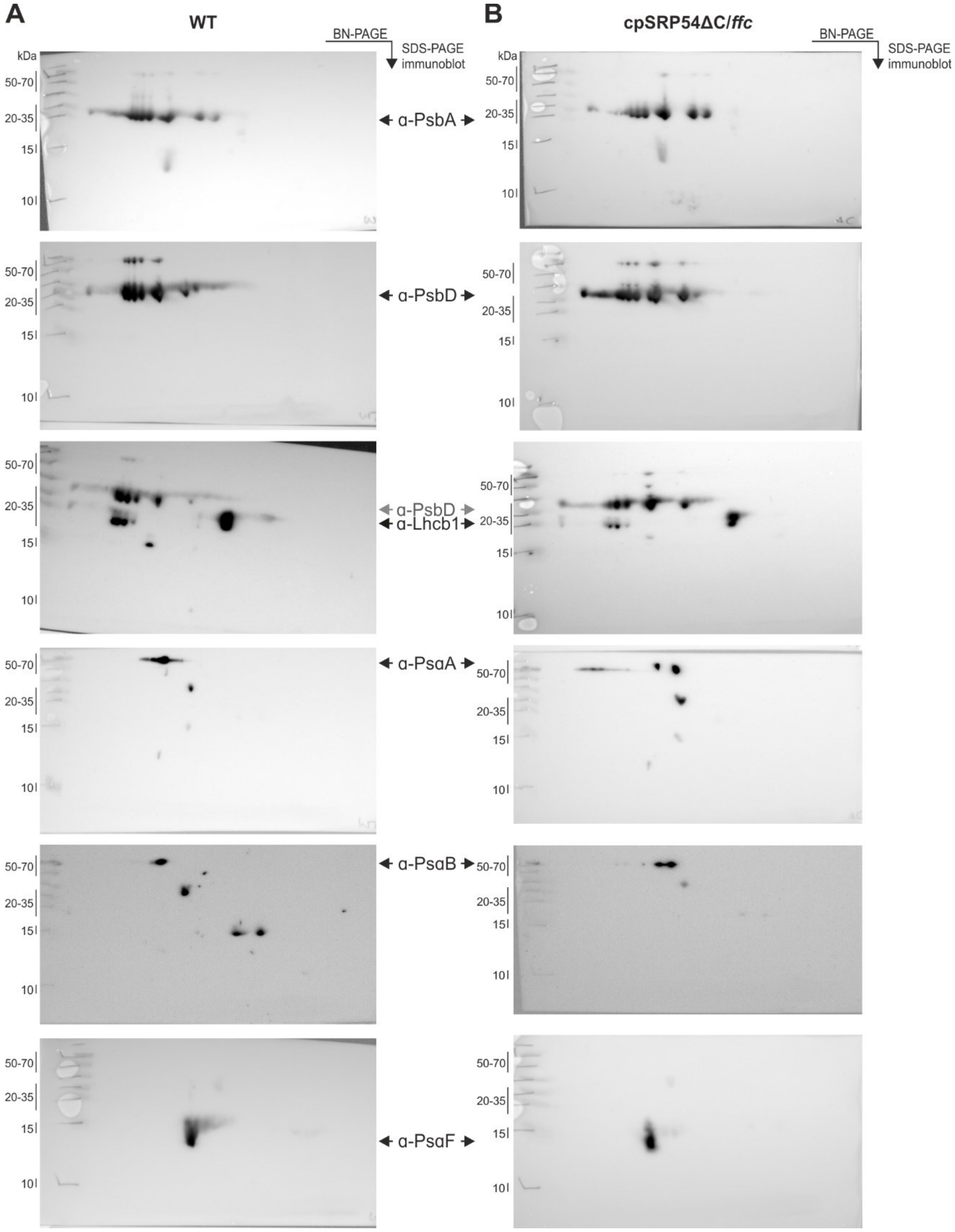

Supplementary Figure S5: Uncropped images of the immunoblots shown in Figure 5.

Uncropped immunoblots of the two-dimensional BN-PAGE/SDS-PAGE analysis of thylakoid membrane multiprotein complexes in *A. thaliana* (**A**) wild type and (**B**) the cpSRP54ΔC/*ffc*-complementation line as shown in Figure 5. Immunoblots are labelled with antibodies against PsbA, PsbD, Lhcb1, PsaA, PsaB and PsaF. Antibody signals labelled in black were used to generate the cropped figures in the main text. Antibodies labelled in grey were previously detected on the same blot membrane. The membranes were then used for further immunodetection.

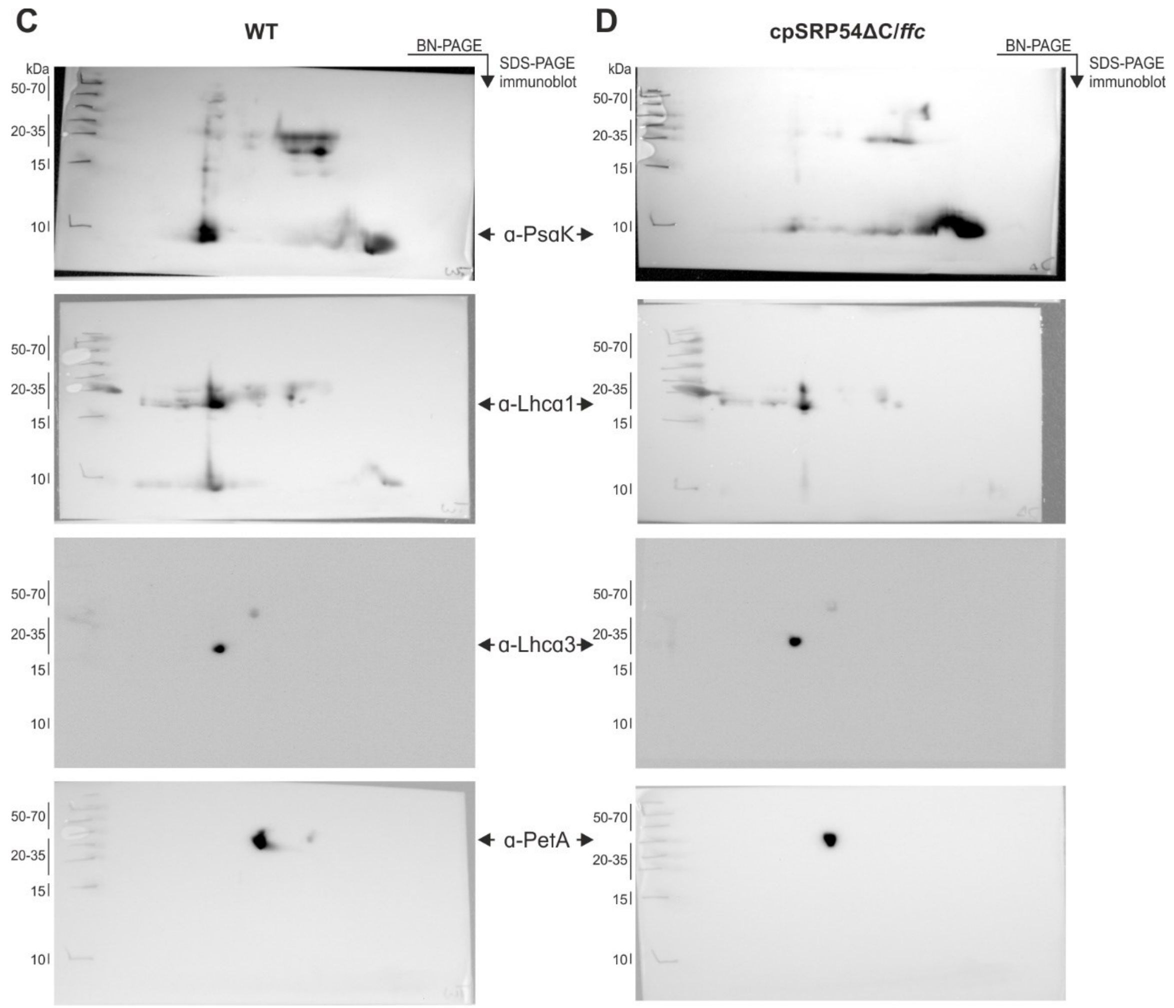

Supplementary Figure S5: continued.

Uncropped immunoblots as shown in Figure 5 depicting the two-dimensional BN-PAGE/SDS-PAGE analysis of thylakoid membrane multiprotein complexes in *A. thaliana* (**C**) wild type and (**D**) the cpSRP54ΔC/*ffc*-complementation line. Immunoblots are labelled with antibodies against PsaK, Lhca1, Lhca3 and PetA.

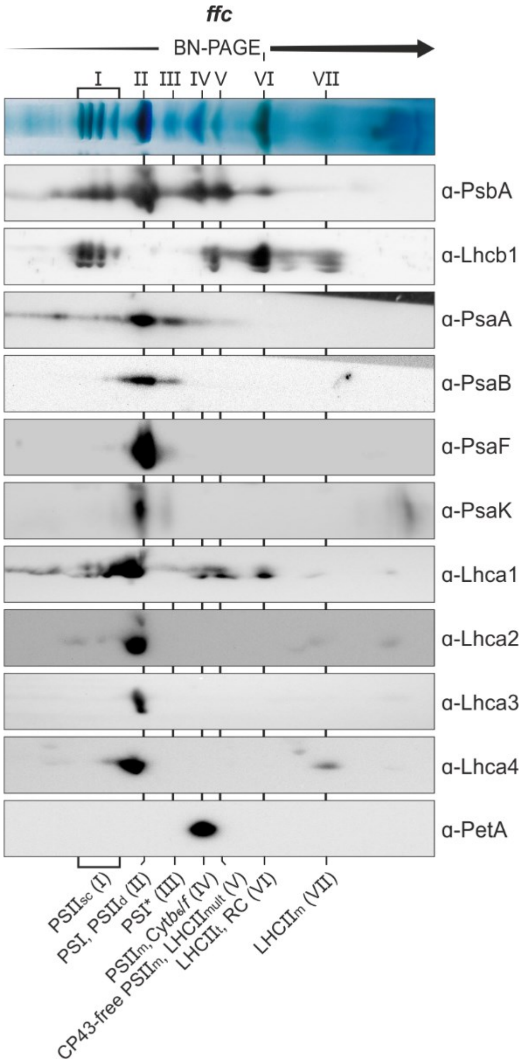

Supplementary Figure S6: Two-dimensional BN-PAGE/SDS-PAGE and immunoblot analysis of thylakoid membrane multiprotein complexes in *A. thaliana ffc.*

Thylakoids of 5-week-old plants were solubilized in DDM and separated by BN-PAGE using 10µg chlorophyll/lane. The identification of detected bands was accomplished in accordance with published BN-PAGE profiles of Arabidopsis thylakoids as mentioned in the main manuscript. Photosystem I and II (PSI and II), PSII supercomplexes ($PSII_{sc}$, I), PSII dimers ($PSII_d$, II), PSI assembly intermediate (PSI*, III), monomeric PSII ($PSII_m$, IV) and cytochrome b_6/f complex (Cytb_6/f, IV), CP43-free PSII monomers (CP43-free $PSII_m$, V) and multimeric light harvesting antenna complex II ($LHCII_{mult}$, V), trimeric LHCII ($LHCII_t$, VI), reaction center-like complex (RC, VI), monomeric ($LHCII_m$, VII). Additionally, the protein complex subunit composition was determined by two-dimensional (2D) BN-PAGE/SDS-PAGE analysis followed by immunoblot analysis with the indicated antibodies.

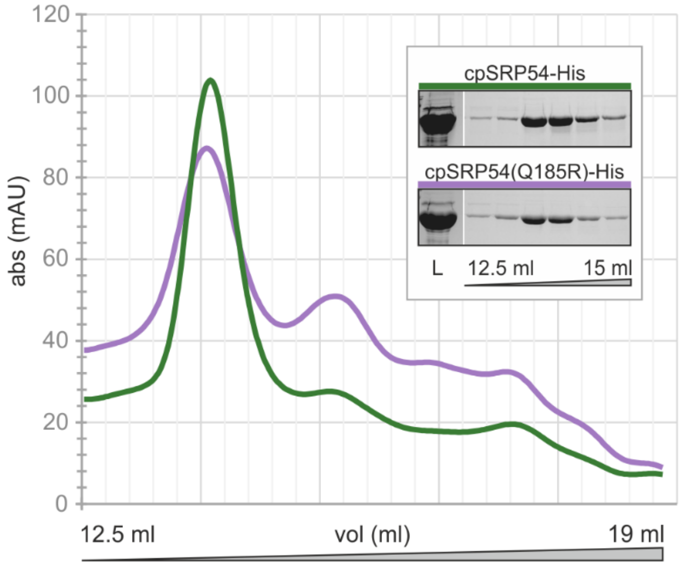

Supplementary Figure S7: Analysis of the migration behaviour of recombinant cpSRP54 and the point mutation variant cpSRP54(Q185R).
Freshly purified recombinant cpSRP54-His and cpSRP54(Q185R)-His were analyzed by size exclusion chromatography (SEC). For cpSRP54-His 2.5 mg and for cpSRP54(Q185R)-His 2.35 mg protein were loaded onto a Superdex200 10/300 GL column (loading sample = L). The SEC was performed using an ÄKTA purifier system.

Supplementary Tables

Supplementary Table S1: Primer used for generation of transformation constructs, site-directed mutagenesis, and genotyping PCRs.

**Primer name**	**Purpose**	**Sequence (5'-3')**
precursor_cpSRP54_XbaI_IFC_pCBi18_for	Generation of transformation construct	GAGAACACGGGGGACTCTAGAATGGAGGCTCTTCAATTTTCC
cpSRP54+His6_SacI_IFC_pCBi18_rev	Generation of transformation construct	CGATCGGGGAAATTCGAGCTCTCAATGGTGATGGTGATGGTGGTTACCAGAGCCGAAGCCACG
cpSRP54NG+His6_SacI_IFC_pCBi18_rev	Generation of transformation construct	CGATCGGGGAAATTCGAGCTCTCAATGGTGATGGTGATGGTGTAGAATTCGTCCAGCCATTCG
cpSRP54ΔC+His6_SacI_IFC_pCBi18_rev	Generation of transformation construct	CGATCGGGGAAATTCGAGCTCTCAATGGTGATGGTGATGGTGCTTTTGTTCTGCTTTCAATGC
At_cpSRP54(Q185R)_for	Site-directed mutagenesis	GTAATATTGTTGGCTGGGCTCCGAGGAGTTGGAAAGACAACGG
At_cpSRP54(Q185R)_rev	Site-directed mutagenesis	CCGTTGTCTTTCCAACTCCTCGGAGCCCAGCCAACAATATTAC
pCBi18_for	Plant genotyping	CCACTGACGTAAGGGATGACGC
pCBi18_rev	Plant genotyping	CATCGCAAGACCGGCAACAGG
At_cpSRP54_gen_for	Plant genotyping	ATGGAGGCTCTTCAATTTTCC
At_cpSRP54_gen_rev	Plant genotyping	CTTGCTTGATGCTGACTCTAC
AL324_cpFtsY-65-BsaI_for	Generation of His6-tagged construct	TTAAGGTCTCCCATGGGCGAGAAAGTTTTCTCCGGATTCTCTAAGAC
AL324_cpFtsY-65-BsaI_rev	Generation of His6-tagged construct	TTAAGGTCTCCTCGAGAGAGAATATAGCATTCACAAAAGCCTCC
AL324_cpSRP54/(Q185R)-Esp3I_for	Generation of His6-tagged construct	TTAACGTCTCCCATGGGCCAGTTGACTGGTGGCCTCG
AL324_cpSRP54/(Q185R)-Esp3I_rev	Generation of His6-tagged construct	TTAACGTCTCCTCGAGGTTACCAGAGCCGAAGCCAC

Supplementary Table S2: Isothermal titration calorimetry (ITC) parameters. Thermodynamic values determined from the ITC experiments.

Experiment (R: run)			K_d (µM)	ΔH (kcal mol^{-1})	-TΔS (kcal mol^{-1})	ΔG (kcal)	N (sites)
**cpSRP54**	**GDP**	**R1**	30.8 ± 5.78	-4.18	-1.98	-6.16	1.55
		**R2**	30.1 ± 6.76	-1.03	-5.14	-6.17	1.04
		**R3**	29.7 ± 3.15	-2.97	-3.20	-6.18	1.79
	**GTP**	**R1**	55.2 ± 9.74	-4.18	-1.63	-5.81	1.47
		**R2**	49.8 ± 4.48	-6.20	0.329	-5.87	1.43
		**R3**	55.3 ± 5.88	-8.66	2.85	-5.81	0.953
**cpSRP54 (Q185R)**	**GDP**	**R1**	30.2 ± 9.12	-4.79	-1.38	-6.17	0.712
		**R2**	30.8 ± 4.28	-7.48	1.33	-6.16	1.46
		**R3**	30 ± 3.97	-5.36	-0.816	-6.17	2.23
	**GTP**	**R1**	17.5 ± 2.86	-4.25	-2.24	-6.49	1.3
		**R2**	17.4 ± 2.06	-8.33	1.84	-6.49	0.936
		**R3**	17 ± 2.1	-8.5	1.99	-6.51	0.863
